# Pressure and stiffness sensing together regulate vascular smooth muscle cell phenotype switching

**DOI:** 10.1126/sciadv.abm3471

**Published:** 2022-04-15

**Authors:** Pamela Swiatlowska, Brian Sit, Zhen Feng, Emilie Marhuenda, Ioannis Xanthis, Simona Zingaro, Matthew Ward, Xinmiao Zhou, Qingzhong Xiao, Cathy Shanahan, Gareth E. Jones, Cheng-han Yu, Thomas Iskratsch

**Affiliations:** 1 School of Engineering and Materials Science, Queen Mary University of London, London, UK.; 2 Randall Centre for Cell and Molecular Biophysics, King’s College London, London, UK.; 3 School of Biomedical Sciences, Hong Kong University, Hong Kong, Hong Kong.; 4 William Harvey Research Institute, Queen Mary University of London, London, UK.; 5 School of Cardiovascular Medicine and Sciences, King’s College London, London, UK.

## Abstract

Vascular smooth muscle cells (VSMCs) play a central role in the progression of atherosclerosis, where they switch from a contractile to a synthetic phenotype. Because of their role as risk factors for atherosclerosis, we sought here to systematically study the impact of matrix stiffness and (hemodynamic) pressure on VSMCs. Thereby, we find that pressure and stiffness individually affect the VSMC phenotype. However, only the combination of hypertensive pressure and matrix compliance, and as such mechanical stimuli that are prevalent during atherosclerosis, leads to a full phenotypic switch including the formation of matrix-degrading podosomes. We further analyze the molecular mechanism in stiffness and pressure sensing and identify a regulation through different but overlapping pathways culminating in the regulation of the actin cytoskeleton through cofilin. Together, our data show how different pathological mechanical signals combined but through distinct pathways accelerate a phenotypic switch that will ultimately contribute to atherosclerotic disease progression.

## Teaser

Pressure and stiffness sensing together regulate VSMC phenotype switching with implications for arterial disease progression.

## INTRODUCTION

Cardiovascular diseases are the primary cause of mortality worldwide. Vascular aging is markedly increasing the risk of cardiovascular disease, including systolic hypertension (HT), coronary artery disease, stroke, heart failure, and atrial fibrillation or atherosclerosis (*1*–*5*). This aging process is related to both chemical/molecular and mechanical changes, including changes to the blood flow (wall stress and hemodynamic pressure) and stiffness of the arterial wall. Endothelial cells (ECs) sense the pressure, wall stress, and shear direction, and the associated mechanosignaling defines atheroprone or atheroprotective regions (*6*, *7*). The shear stress deforms the apical surface of ECs and is sensed among others by the glycocalyx, mechanosensitive ion channels, and various receptors including PECAM-1, plexin D1, and integrins (*6*, *8*–*11*). ECs communicate this then further to the underlying arterial layers and the residing vascular smooth muscle cells (VSMCs) through the extracellular matrix, growth factor signaling, and direct receptor interaction (*12*).

VSMCs are located in the media layer and are necessary for matrix formation and contractility of the arterial wall. In response to pathological signals, they migrate into the intima layer and adopt alternative phenotypes, which have been described as synthetic, macrophage-like, or foam cells (*13*). Previous studies on the involvement of VSMCs in atherosclerosis have largely focused on their role in advanced atherosclerotic processes, i.e., fibrous cap formation and plaque stability or rupture. However, recent research increasingly points to a critical role for VSMCs in the initiation and early phases of atherosclerosis, including diffuse intimal thickenings—the most likely precursor of pre-atherosclerotic plaques (*13*, *14*), as well as pathological intimal thickening, which is considered the first stage of atherosclerosis (*13*). In diffuse intimal thickenings, VSMCs are considered the major source of extracellular matrix proteins and especially proteoglycans (versican and biglycan, as well as others to a lesser degree) in the arterial wall, hence leading to the thickening of the intima and the progression to pathological intimal thickening (*13*, *15*, *16*). This progression is promoted by retention of apolipoproteins, oxidation of lipids, VSMC phenotypic switching, proliferation, and apoptosis. Again, the capability of (phenotypically altered) VSMCs to secrete and remodel the extracellular matrix is critical for this step (*17*, *18*).

In addition to the role of EC-VSMC cross-signaling, there is circumstantial evidence suggesting that mechanical forces also directly impact on the VSMC phenotype. Diffuse intimal thickenings have long been considered an adaptation to blood flow (*19*). Moreover, mechanical strain altered mRNA expression and accumulation of extracellular matrix proteins from VSMCs (*20*). Lastly, pressure induced phenotypic changes and migration in VSMCs (*21*, *22*)—although these studies used static pressure and rigid tissue culture plastic and therefore nonphysiological stimuli.

Arteries are composite structures, with mechanical properties that differ between micro- and macroscale, and direction of strain. Generally, microscale stiffnesses are magnitudes lower compared to the macroscale (*23*, *24*), which is a result of the specific mechanical behavior at different length scales, as well as sample preparation and measurement techniques (*25*). At the microscale, comparative studies between the individual arterial wall layers suggested that the intima was more compliant compared to the media layer (*26*) and studies report values that ranged from 5 to 50 kPa (*26*, *27*) and ~30 to 190 kPa (*28*–*30*) in the intima and media layers, respectively. In addition, measurements of lipid-rich regions in (human and ApoE^−/−^ mouse) atherosclerotic plaques indicated a compliant environment (2.7 ± 1.8 kPa and 5.5 ± 3.5 kPa, respectively) and potentially further softening at the microscale during diffuse or pathological intimal thickenings where proteoglycan and lipid content increases (*27*, *31*).

The expression of proteoglycans not only affects the compliance but also results in a larger proportion of void space and, hence, increased compressibility of the intima and the residing VSMCs. VSMCs are poro-elastic in nature. The entry and exit of water through aquaporins is further enabled by the elastic network of the cytoskeleton, which deforms due to pressure, tension, and shearing stresses (*32*–*34*). Conclusively, previous studies suggested that the compression of the intima increases (*35*), while its stretching decreases due to the accompanying arterial macroscale stiffening (*23*, *24*, *36*, *37*).

Because of the changing mechanical stimuli in aging and vascular disease, we sought here to study the effects of the different mechanical stimuli (pressure, stretch, and rigidity) in isolation to identify critical parameters influencing phenotypic changes of the VSMCs. Thereby, we find individual effects of pressure and compliance, which both favor a phenotypic switch. This switch is potentiated when combining compliance and pressure. In combination of these two stimuli, we find large-scale changes to protein expression, cell morphology, actin organization, and maximal formation of matrix-degrading podosomes. We further study the regulation of the podosomes downstream of mechanical signals and identify distinct molecular pathways, both acting on the same protein, cofilin, which regulates the podosome formation and turnover. Together, our data highlight the strong contribution of different mechanical factors on VSMCs that, in combination, will influence atherosclerotic disease progression.

## RESULTS

### The phenotypic switch of VSMCs is regulated through hemodynamic pressure and matrix stiffness

**Fig. 1.**
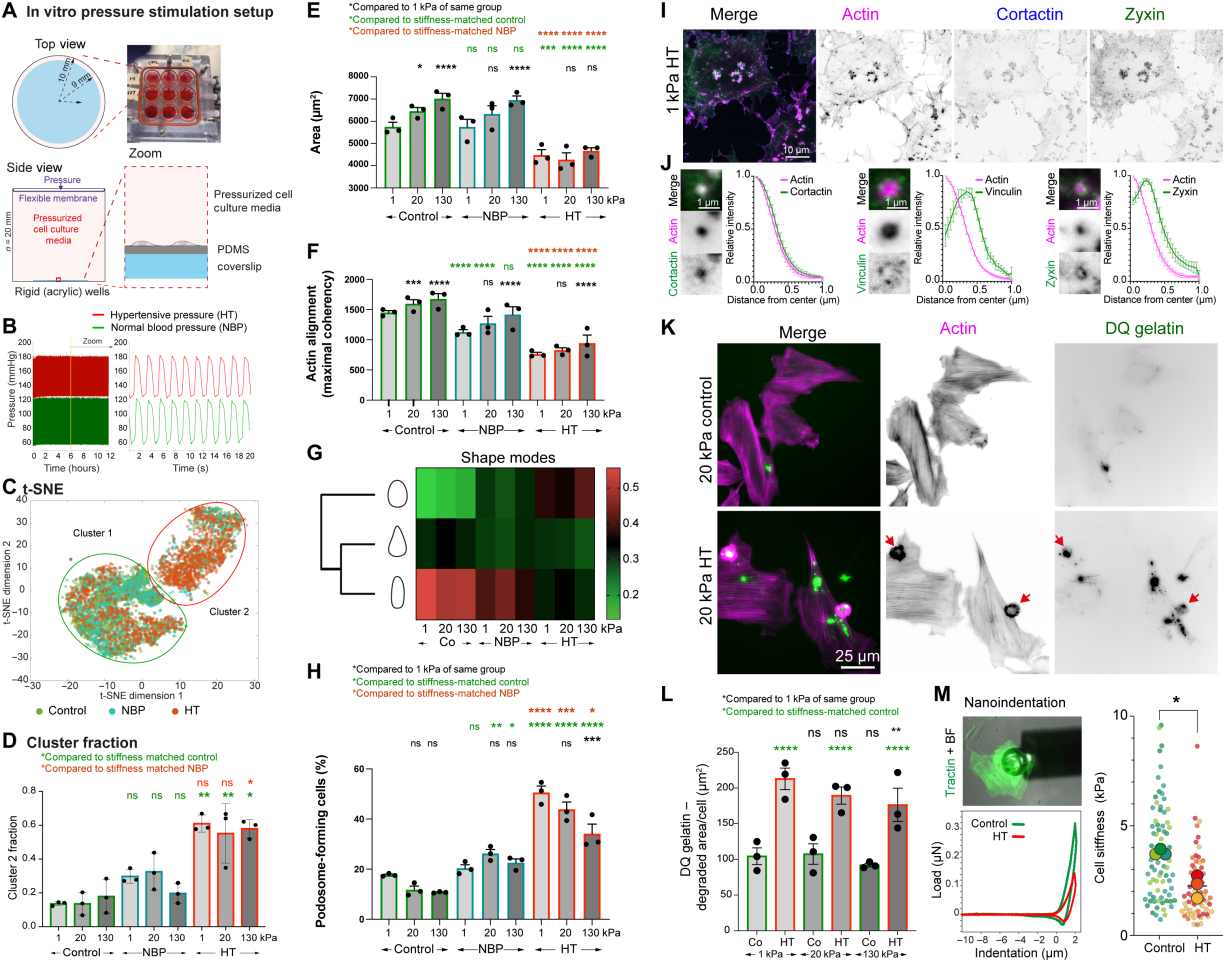
HT stimulates phenotypic switching in VSMC. (**A**) Overview of the experimental setup and (**B**) pressure settings. (**C** and **D**) Cells were analyzed using CellProfiler before dimensionality reduction and cluster analysis (t-SNE). (**E**) HT treatment reduced cell area, (**F**) actin alignment (**G**), and shape of the cells. (**H**) The fraction of podosome-forming cells depended on pressure and stiffness (displayed as average per repeat with ~150 to 200 cells each). (**I**) Podosomes were confirmed by costaining for cortactin, zyxin, or vinculin. (**J**) Radial profiles are normalized intensities from *n* = 10 podosomes from five cells each. (**K** and **L**) Gelatin degradation (DQ gelatin–positive area) per cell, displayed as average per repeat, from *n* = 200 to 300 cells each. (**M**) The large-scale changes to the cellular cytoskeleton were also reflected by a drop in the cellular stiffness as determined by nanoindentation. Top left: Image from tractin (green) and the indenter (bright-field channel). Bottom left: Typical load-indentation curve for the control (green) and HT (red)–treated cells. Right: Young’s moduli [graph from SuperPlotsOfData (*78*)]. *P < 0.0332; **P < 0.0021; ***P < 0.0002; ****P < 0.0001; ns, not significant from ANOVA test with correction for multiple comparisons (D to F, H, and L) or *P < 0.05 from unpaired Welch’s *t* test (M).

VSMCs play a key role in various stages of atherosclerosis, and disease progression is associated with phenotypic switching. Extracellular matrix changes and hypertensive blood pressure are both critically linked to the onset and progression of atherosclerosis. Therefore, we wanted to study the effect of the different mechanical stimuli (rigidity, pressure, and stretch) on VSMC phenotype. Specifically, we plated A7r5 VSMCs onto PDMS-coated coverslips with defined stiffness covering the whole range that VSMCs are exposed to, from an intima with enhanced proteoglycan expression and lipid inclusions (1 kPa) (*27*, *31*) to healthy intima layer (20 kPa) (*26*, *27*) and maximal stiffness values reported for the media layer, or calcified regions in the atherosclerotic plaques (130 kPa) (*27*–*31*). In addition, the VSMCs on the stiffness-defined substrates were subjected to hydrodynamic pressure stimulation, mimicking either healthy normal blood pressure (NBP; 120/60 mmHg) or stage II HT (180/120 mmHg) (Fig. 1, A and B), using a pressure stimulator (CellScale Mechanoculture TR, modified for a low-pressure range), situated inside a 37°C tissue culture incubator. Control cells were kept inside the chamber without the pressure stimulation. After 12 hours, cells were fixed and stained with phalloidin before cell segmentation and analysis of morphological parameters with CellProfiler (cell shape and F-actin shape descriptors) and the ImageJ OrientationJ plugin (F-actin alignment) (*38*, *39*). To detect F-actin dots, which we initially observed in some cells, we trained a pixel classification using Ilastik and measured the number and the area of dots in cells after the segmentation. We then performed a dimensionality reduction using *t*-distributed stochastic neighbor embedding (t-SNE) (Fig. 1C), and two separate clusters were identified, including primary control and NBP-treated cells (cluster 1) or HT-treated cells (cluster 2) (Fig. 1D). Overall, we noted a correlation of the cluster composition with the pressure stimulation regime and, to a smaller degree, with the substrate stiffness (Fig. 1D). After HT treatment, we also found a substantial change to the cell area (Fig. 1E) and actin organization, including a reduction in actin alignment and overall organization (Fig. 1F). Consistent with this, cell shape analysis using the visually aided morphophenotyping image recognition “VAMPIRE” software (*40*) indicated an increase in round-shaped compared to spindle-shaped cells after the HT treatment (Fig. 1G), while NBP treatment in contrast preserved spindle shapes.

VSMCs are known to assemble podosomes in response to chemical or biophysical stimuli. VSMC podosomes have also been identified in vivo and are a hallmark of phenotypic switching (*24*, *41*, *42*). Podosomes consist of a core of densely polymerized F-actin, surrounded by a ring complex, containing adhesion proteins. We also detected changes to actin dot occurrence, which occurred either as single dots or clusters and were identified as podosomes (Fig. 1, H to J). Using superresolution spinning disc microscopy, we found that cortactin, one of the podosome markers, localized with the F-actin core in VSMCs, which was surrounded by adhesion proteins zyxin or vinculin (Fig. 1, I and J). Localized degradations of the gelatin matrix were also observed around the podosome (Fig. 1, K and L). Unexpectedly, the number of podosome-forming cells not only showed a strong increase after pressure stimulation compared to the control but also depended largely on matrix stiffness, with a higher number of podosome-forming cells on compliant surfaces. To confirm this unexpected result, we examined the effect of pressure and stiffness on primary bovine and human cells, which all recapitulated the observed changes (fig. S1). Noticeable was a blunted effect on one human cell isolate due to a higher podosome-forming activity under control conditions. Because these cells were already cultured for >10 passages, this was likely due to onsetting dedifferentiation and senescence. In terms of matrix remodeling, there were more podosome-forming (A7r5) cells and greater gelatin degradation on the 1 kPa surface, compared to 130 kPa (Fig. 1, K and L). The extensive changes to the cytoskeletal structures were accompanied by a reduction in the cellular stiffness (Fig. 1M; mean ± SEM: 3.78 ± 0.24 kPa versus 2.21 ± 0.18 kPa in control and HT-treated cells). The measurement was performed by nanoindentation with a large-diameter spherical tip, placed approximately above the nucleus (tip radius: 50 μm, *k* = 0.5 N/m), and the data are therefore a combination of both actin cytoskeleton and nuclear stiffness (*43*).

**Fig. 2.**
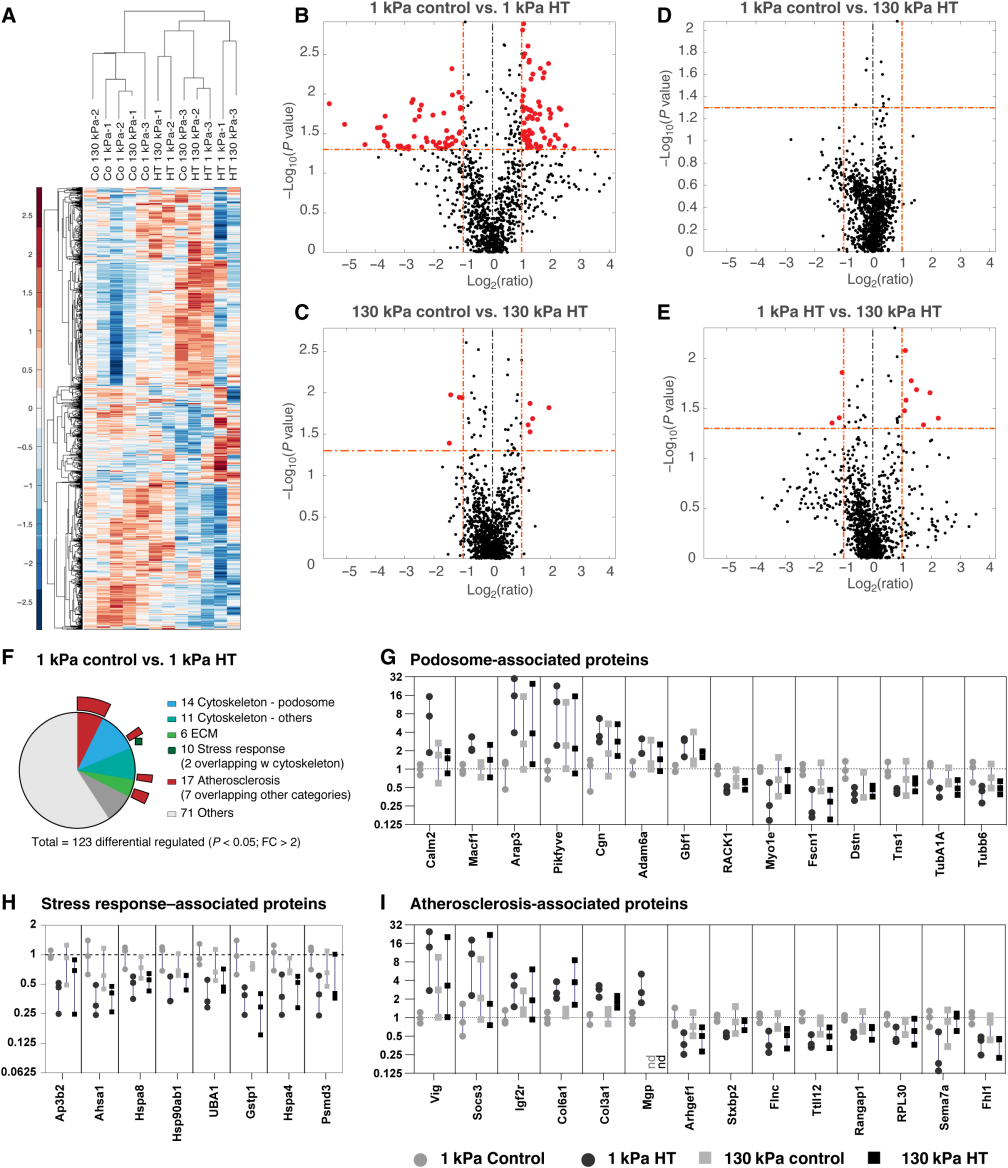
Quantitative proteomics analysis indicates stiffness- and pressure-dependent changes. (**A**) Hierarchical clustering of identified proteins indicates primary clustering of HT-treated and control A7r5 VSMCs. Pairwise differential regulation analysis indicates 123 significant changes (FC > 2, *P* < 0.05; shown in red) between 1-kPa control and HT-treated samples (**B**), compared to no or only few significant changes for the other comparisons (**C** to **E**). (**F** to **I**) Literature and gene ontology analysis of the identified 123 differential regulated proteins identified 14 proteins associated with podosome formation (G), 11 other cytoskeletal proteins, 6 extracellular matrix (ECM) components, 10 proteins associated with stress response (H), and 17 proteins associated with atherosclerosis (I) [numbers including overlaps, see (F), outer circle; (G to I) α and β tubulin as well as Myo1e are shown in (G), but are also associated with atherosclerosis; α and β tubulin also with stress response]. From left to right: Gray dots: 1-kPa control, black dots: 1-kPa HT, gray squares: 130-kPa control, black squares: 130-kPa HT.

To further investigate the different behaviors of VSMCs, we performed quantitative proteomics analysis on soft (1 kPa) or stiff (130 kPa) surfaces in the presence or absence of cyclic hypertensive pressure. Abundance of smooth muscle proteins (including smooth muscle actin, metavinculin, caldesmon 1, and calponin) confirmed the retention of a smooth muscle phenotype of the A7r5 cells (table S1). Hierarchical analysis of 1026 quantified proteins indicated primarily clustering of pressure-treated samples versus control samples (Fig. 2A). Individual pairwise analysis identified no or only few significant changes between 1 kPa control versus 130 kPa control, 1 kPa HT versus 130 kPa HT, or 130 kPa control versus 130 kPa HT treated samples. In contrast, the pairwise comparison between 1 kPa control and 1 kPa HT pressure-treated sample indicated 123 proteins that were significantly up- or down-regulated [fold change (FC) > 2, *P* < 0.05] (Fig. 2, B to E, and tables S2 to S5). The differential regulated proteins included 14 podosome-associated proteins, 9 other cytoskeletal proteins, 10 stress response proteins (all down-regulated with pressure stimulation), and 17 proteins that were previously associated with atherosclerosis or neointima formation (Fig. 2, F to I). Together, these results suggest that the HT pressure globally alters the cell morphology and the actin organization, but cells on the softer surface mimicking the stiffness of (pre-)atherosclerotic lesions show a distinct increase in the formation of matrix-degrading podosomes.

### Substrate stretching is insufficient to induce the podosome formation

The hemodynamic pressure results in compression and stretching of the extracellular matrix and cells in the arterial wall. The distension along the inner circumference of most human arteries was measured between 5 and 10% (*35*, *44*, *45*), whereby the distension decreases in hypertensive patients, because of the accompanying arterial macroscale stiffening (*46*). Therefore, we applied a 10% cyclic biaxial stretch regime at the same frequency and duration of the pressure experiment (0.5 Hz for 12 hours) to test whether stretch alone is sufficient to induce the observed changes. After analyzing the VSMCs with the same image analysis pipeline (see Materials and Methods), we found, however, no clear differences between the cell populations (fig. S2, A and B). Although we detected a small but significant decrease of the cell area (fig. S2C), we did not detect any other changes to cell shape, actin organization, or the ability to form podosomes (fig. S2, D to G). Similarly, 30-min static pressure application (180 mmHg) stimulated podosome formation with a strong stiffness dependence, but we detected no change in the ability to form podosomes after 30-min static biaxial stretch (5 or 10%; fig. S3).

### Vascular smooth muscle podosomes are mechanosensitive after induction through chemical stimuli

**Fig. 3.**
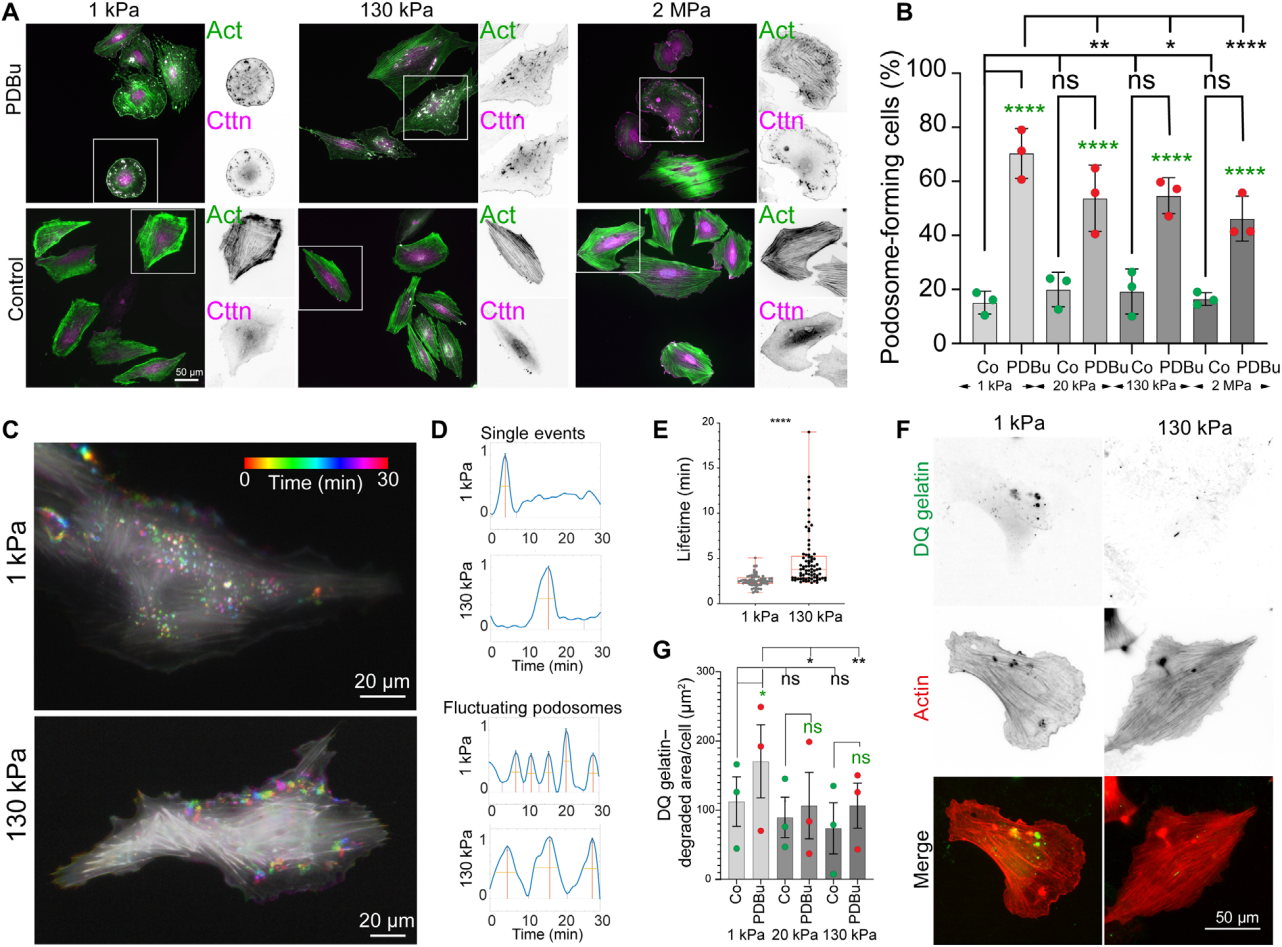
PDBu treatment leads to efficient podosome formation on all stiffnesses, but podosome behavior and matrix degradation are controlled by stiffness. (**A** and **B**) PDBu treatment leads to stiffness-dependent podosome formation in A7r5 VSMCs (fraction of podosome-forming cells, displayed as average per repeat, from three independent experiments with ~100 to 200 cells each). (**C** and **D**) Podosome lifetime depends on substrate stiffness. (C) Color-coded time projection of A7r5 VSMC treated with PDBu on 1 and 130 kPa shows dynamic podosome formation (see also movies S1 and S2). (D and **E**) Podosome formation (independent of stiffness) was detected as either single event or cyclically fluctuating podosomes but showed stiffness dependence in their turnover time (measured at half-maximum of the peaks) with an increased lifetime on higher stiffness (E). (**F** and **G**) Gelatin degradation was more extensive on compliant surfaces (DQ gelatin–positive area per cell, displayed as average per repeat, from three independent experiments with *n* = 30 to 100 cells analyzed per repeat and condition). *P < 0.0332; **P < 0.0021; ***P < 0.0002; ****P < 0.0001; *P* values from ANOVA and Bonferroni test for multiple comparisons or unpaired *t* test (E); (G) green asterisks represent comparison to the controls of same stiffness group, and black asterisks represent comparison to 1 kPa of same treatment group.

In addition to hypertensive blood pressure, disturbed flow and EC-VSMC cross-talk through various protein kinase C (PKC)–activating signals from the ECs are powerful stimuli for intima lesion formation and atherogenesis (*12*). Phorbol 12,13-dibutyrate (PDBu), a potent activator of PKC signaling (*47*), has been previously used to induce the formation of podosomes in VSMCs (*48*). Consistently, we found a strong induction of podosome formation after PDBu treatment (Fig. 3, A and B). In particular, we detected a significantly higher fraction of podosome-forming cells, as well as significant differences in the turnover and function of podosomes on the substrate with lower stiffness (Fig. 3, C to E). Over time, podosomes were forming and turning over only once, or frequently cyclically appearing and disappearing at the same location (fluctuating podosomes; Fig. 3, C and D, and movies S1 and S2). Moreover, podosomes often appeared in groups, whereby the formation and disappearance followed a wave-like pattern, suggesting the cross-talk and time-delayed coordination among the podosomes. While we did not detect an obvious correlation between such wave pattern and stiffness, we found that podosomes on the soft surfaces turned over more rapidly (Fig. 3E) and were degrading the extracellular matrix more efficiently on soft surfaces compared to cells plated on higher stiffness (Fig. 3, F and G). Together, these results suggest that PDBu treatment leads to highly efficient activation of podosome formation, but the stiffness-dependent behavior persists.

### Podosomal actin turnover in VSMCs is controlled by substrate stiffness

**Fig. 4.**
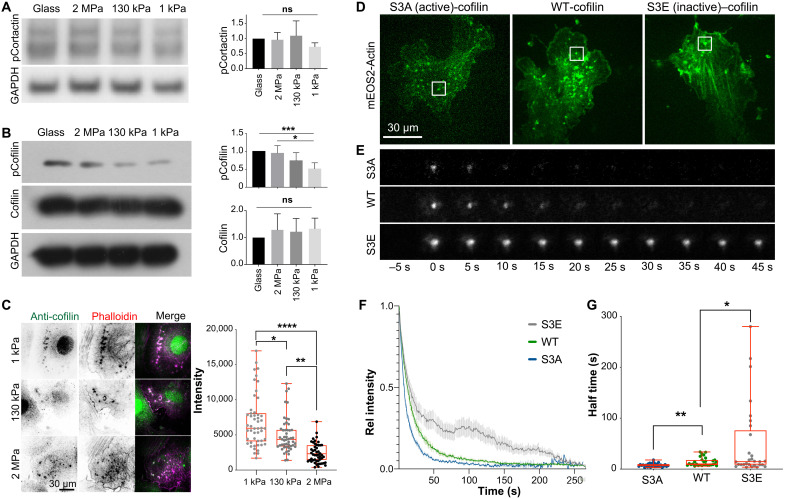
Cofilin localization and activity respond to stiffness and regulate podosomal actin turnover. (**A**) Cortactin phosphorylation is unaffected by stiffness, but cofilin phosphorylation is reduced on soft surfaces, indicating higher activity. Error bars: SD; test for linear trend between stiffness and pCofilin levels: *P* = 0.0001. (**B**) Cofilin is enriched at podosomes on soft surfaces. (**C**) Transfection of phosphomimicking or nonphosphorylatable cofilin regulates the turnover of podosomal actin. (**D**) A7r5 VSMCs were transfected with mEOS2-actin and iRFP-cofilin WT, S3A (active), or S3E (inactive). (E) Actin turnover was measured after photoswitching of mEOS2-actin, indicating changes in lifetime. (**E** to **G**) Fluorescence loss after photoconversion indicates slower podosomal actin turnover in the presence of S3E and faster turnover in the presence of S3A cofilin mutants. Plot of mean and SEM of 30 podosomes from 10 cells per condition (F) and half-time (G). *P < 0.0332; **P < 0.0021; ***P < 0.0002; ****P < 0.0001; *P* values from ANOVA and Bonferroni test. Box plots are displayed as median, upper and lower quartile (box), and range (whiskers).

Faster turnover and recurrence of podosomes on compliant substrates suggested a stiffness-dependent F-actin assembly. F-actin assembly at the podosome core is regulated through cortactin (via N-WASP and Arp2/3), or alternatively the actin-severing protein actin depolymerizing factor (ADF)/cofilin, which accelerates actin dynamics by generating additional barbed ends and has reported actin filament nucleation activity at high concentrations (*49*). The activities of cortactin and ADF/cofilin are regulated by the phosphorylation at Y421 and S3, respectively. We performed Western blotting with phospho-specific antibodies and found that levels of phosphorylated Y421 cortactin did not change with stiffness. However, there was a significant decrease in cofilin S3 phosphorylation on lower stiffnesses, suggesting a higher level of cofilin activity that was consistent with increased podosome dynamics (Fig. 4, A and B). In addition, a stronger enrichment of cofilin at the podosome core was observed on softer surfaces (Fig. 4C). To confirm the relevance of cofilin phosphorylation, we cotransfected mEOS2-actin and phosphorylation mutants of cofilin and measured the impact on the turnover of podosomal actin. After spot photoactivation, the dynamics of photo-converted mEOS2-actin at the podosome were monitored over time. Here, the presence of an active, nonphosphorylatable, cofilin mutant (S3A) increased actin turnover, while the presence of a phosphomimicking S3E cofilin strongly reduced the turnover (Fig. 4, D to G). Together, these data suggest that cofilin is instrumental in regulating podosomal actin turnover downstream of extracellular matrix stiffness sensing.

### Podosomal cofilin is regulated through RhoA, ROCK2, and LIMK2 in a stiffness-dependent manner

**Fig. 5.**
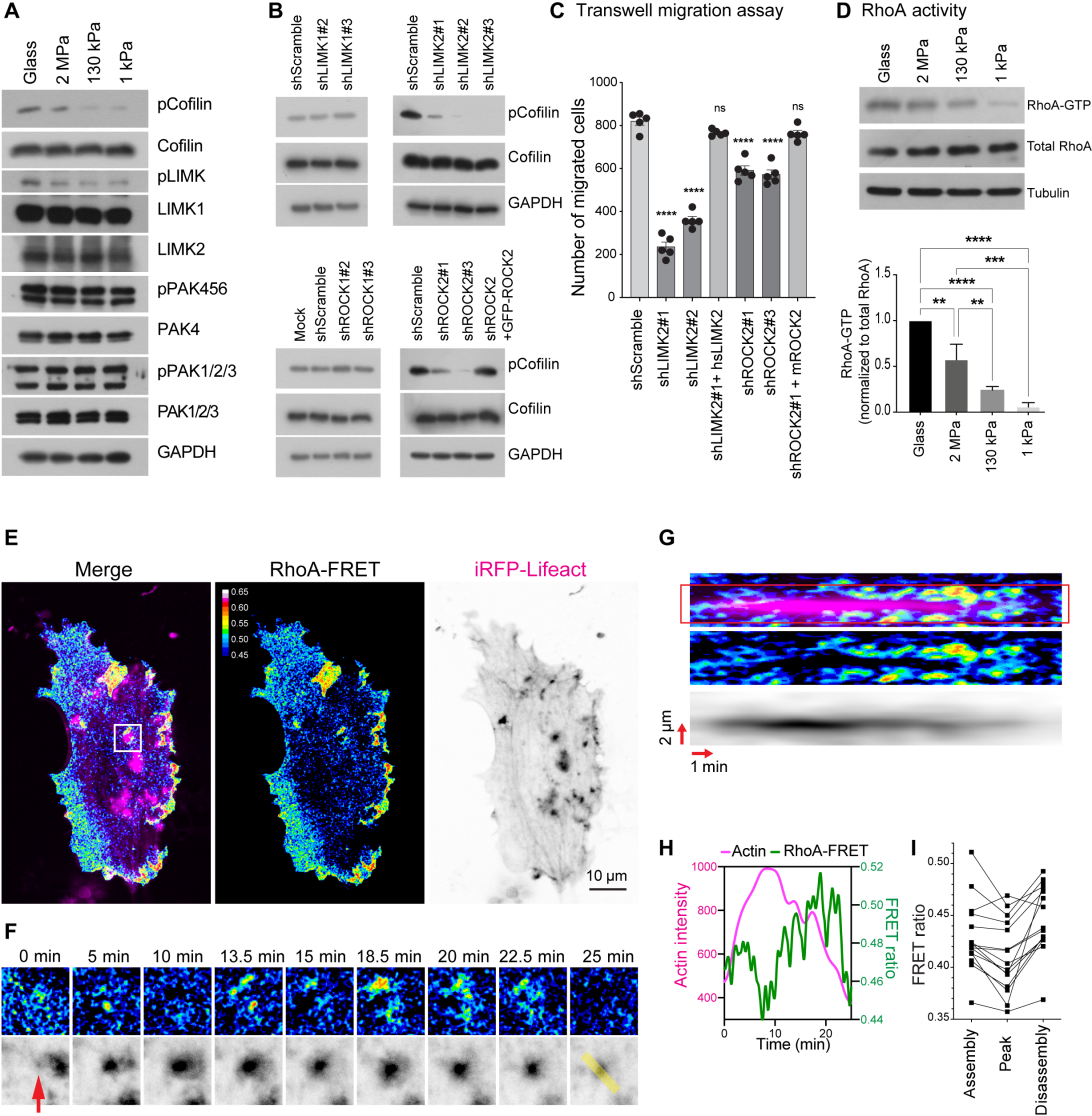
Rigidity sensing controls cofilin activity through RhoA-ROCK-LIMK signaling. (**A**) WB analysis of A7r5 VSMCs seeded on different stiffnesses. pCofilin and pLIMK are reduced on compliant surfaces, while other antibodies do not detect stiffness-dependent changes. (**B**) LIMK2 and ROCK2 knockdown, but not LIMK1 or ROCK1 knockdown, reduces pCofilin levels in A7r5 VSMCs (see fig. S4 for knockdown validation). (**C**) LIMK2 and ROCK2 knockdown reduces transwell migration of VSMCs. Error bars: SEM. (**D**) RhoA-GTP pull-down assay shows a reduction in active RhoA on compliant surfaces (quantification from three independent repeats; error bars: SD). (**E** to **G**) A7r5 cells were transfected with a RhoA-FRET biosensor and iRFP-Lifeact to analyze the dynamic changes to RhoA activity after PDBu stimulation. Cyan fluorescent protein (CFP)/yellow fluorescent protein (YFP) images were taken every 5 s after donor excitation with a 445-nm laser and with a dual camera setup for simultaneous acquisition (see also fig. S7). iRFP images were taken every 100 s as a reference for the podosome position. (F) Time points of area outlined in white in (E). (G) Kymograph over line marked in yellow in (F). (**H**) Intensity profiles over time of a 3-μm-wide line marked by outlines in (G). (**I**) FRET intensity at half-maximum actin intensity during assembly and disassembly as well as at peak intensity from *n* = 15 podosomes from three independent experiments. The iRFP image stack was filtered with a 3D Gaussian filter (*x* = 1, *y* = 1, *z* = 10) for visualization purpose in (G) and (H). *P < 0.0332; **P < 0.0021; ***P < 0.0002; ****P < 0.0001; *P* values from ANOVA and Bonferroni test (C and D).

LIM domain kinases (LIMKs) are known to directly phosphorylate cofilin at S3 and to reduce its actin severing activity (*50*). The activation of LIMK can be positively regulated by ROCK- or PAK-mediated phosphorylation. We sought to investigate how cofilin phosphorylation is regulated downstream of mechanical stimuli. In PDBu-stimulated VSMCs, we found that the level of phosphorylated LIMK (T508 in LIMK1 and T505 in LIMK2) showed the same trend as that of phosphorylated cofilin, and higher levels of phosphorylation were observed on the stiff surface (Fig. 5A). Because the phosphoantibody detects both LIMK1 and LIMK2, we knocked down each isoform and found that LIMK2 was responsible for phosphorylating cofilin in A7r5 VSMCs (Fig. 5B and fig. S4 for knockdown validation). Next, we investigated the potential upstream kinases to promote the LIMK phosphorylation. We found that the activities of group 1 PAK (PAK1/2/3) and group 2 PAK (PAK4/5/6), detected by the respective phospho-specific antibodies, did not demonstrate any stiffness dependence. However, when ROCK1 and ROCK2 were knocked down individually, we found a strong reduction in cofilin phosphorylation after the knockdown of ROCK2 (Fig. 5, A and B, and fig. S4 for knockdown validation). In agreement with the effect of the knockdowns, pan-inhibitions of Rho kinases with Y27632 (20 μM) and H1152 (10 μM) or ROCK2-specific inhibition with KD025 (10 μM) resulted in the decrease of both cofilin and LIMK phosphorylation (fig. S5). In addition, we found that both LIMK2 and ROCK2 were colocalized and specifically enriched at the VSMC podosome (fig. S6). When ROCK2 and LIMK2 were individually knocked down, the transwell migration of PDBu-treated VSMCs was strongly reduced (Fig. 5C). Reintroductions of ROCK2 and LIMK2 in the respective knockdown condition restored the migration capability.

RhoA is known to act as an upstream messenger to activate ROCK-mediated signal transductions. We further identified higher proportions of guanosine 5′-triphosphate (GTP)–bound RhoA when VSMCs were plated on the stiff substrate (Fig. 5D). Using a RhoA fluorescence resonance energy transfer (FRET) activity sensor, we found high RhoA activity in areas surrounding the podosome core (Fig. 5, E to H; fig. S7; and movie S3). Initially, the RhoA activity was reduced during the podosome assembly, as predicted previously (*51*, *52*). However, flashes of RhoA activity increasingly and consistently appeared in the vicinity of the podosome core during the podosome disassembly (Fig. 5, F to I).

Apart from RhoA, Cdc42 is also reported to promote cofilin phosphorylation through PAK-mediated LIMK phosphorylation (*53*). VSMCs with Cdc42-GTP inhibition by ML141 (10 μM) exhibited a lower level of cofilin phosphorylation but did not show the deceased phosphorylation of PAK and LIMK, suggesting a noncanonical effect of Cdc42 inhibition on cofilin (fig. S5). Together, these results show stiffness-dependent and dynamic changes to RhoA activity, which regulate ROCK2- and LIMK2-mediated cofilin phosphorylation.

**Fig. 6.**
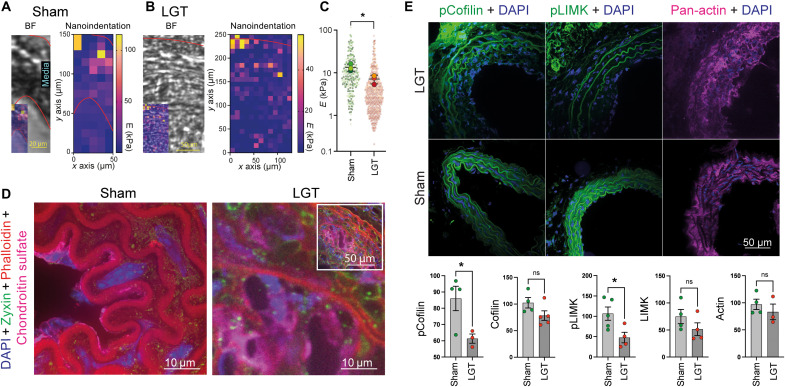
Neointima formation is accompanied by reduced extracellular matrix stiffness and changes to LIMK and cofilin phosphorylation. (**A** to **C**) Nanoindentation measurements indicate a reduced elastic modulus during neointima formation. (A and B) Bright-field (BF) images before nanoindentation (inset: overlay with measured Young’s modulus) for sham-treated mice (A) or after carotid artery ligation (B: LGT). Quantified in (C), graph from SuperPlotsOfData (*78*) with *P* < 0.05 from Welch’s *t* test for comparison of means between conditions. (**D**) The increased compliance coincides with increased proteoglycan expression (chondroitin sulfate staining). Inset for LGT shows larger area for overview. (**E**) Frozen sections from sham-treated mice and animals that underwent carotid artery ligation, stained for pCofilin, pLIMK, and pan-actin together with DAPI. Quantification of the staining intensities of mouse VSMC in sham and ligation (LGT) samples indicates a reduction of both pCofilin and pLIMK, while actin, cofilin, and LIMK intensities are unaffected. Only cell areas were included in the quantification, which was not affected by typical background staining from the elastin layers. Data presented as mean per animal from *n* = 221 and 163 cells (sham and LGT, respectively, pCofilin), 274 and 330 cells (cofilin), 288 and 142 cells (pLIMK), 195 and 238 cells (LIMK), and 311 and 246 cells (actin); *P < 0.0332; *P* values from unpaired *t* test (C and E).

Next, we sought to examine the link between matrix rigidity, LIMK, and cofilin phosphorylation in vivo. Previous studies, examining lipid-rich regions in atherosclerotic plaques, indicated that the intima might also undergo softening during its remodeling and neointima formation (*27*, *31*). Mouse carotid artery ligation is a well-established model for intimal lesion formation, whereby disturbed flow pattern and endothelial mechanosensing lead to neointima formation (*54*). We first assessed the mechanical properties of sham-treated and carotid ligation tissues (LGT) by nanoindentation. Because the nanoindentation setup (500-nm indentations with a probe with radius = 50 μm and *k* = 0.5 N/m) resulted in a contact diameter of ~10 μm, we spaced the individual measurements 10 μm in radial direction and axial direction. Subsequently, we aligned the measurements with the bright-field image to assign the stiffness map with the arterial wall area of the tissue sections (Fig. 6, A to C).

Our measurements (total of *n* = 220 and 433 indentations from three animals each for sham and LGT) indicated in both cases a stiffness gradient from the luminal side to the outside of the artery. Overall, the measurements confirmed a reduced elastic modulus in the LGT model (mean ± SEM: 14.6 ± 1.3 kPa versus 6.0 ± 0.4 kPa; Fig. 6, A to C), coinciding with an increase in proteoglycan content as indicated by chondroitin sulfate staining (Fig. 6D). While the nature of the samples did not allow us to identify the presence of podosomes, we nevertheless detected an accumulation of actin puncta and surrounding adhesion (zyxin) structures (Fig. 6D and fig. S8), consistent with previous reports of podosome formation in VSMCs in vivo (*41*). We also detected a reduction in phospho-cofilin and phospho-LIMK staining in VSMCs in the neointima, while the actin, cofilin, and LIMK staining intensities remained unaffected (Fig. 6E). Together, our results show stiffness-dependent and dynamic changes to RhoA activity, which regulate ROCK2- and LIMK2-mediated cofilin phosphorylation in vitro, and a correlation between stiffness and LIMK and cofilin phosphorylation in VSMCs in vivo.

### Pressure and PDBu stimulate cofilin dephosphorylation and podosome formation through Ca^2+^ and SSH

**Fig. 7.**
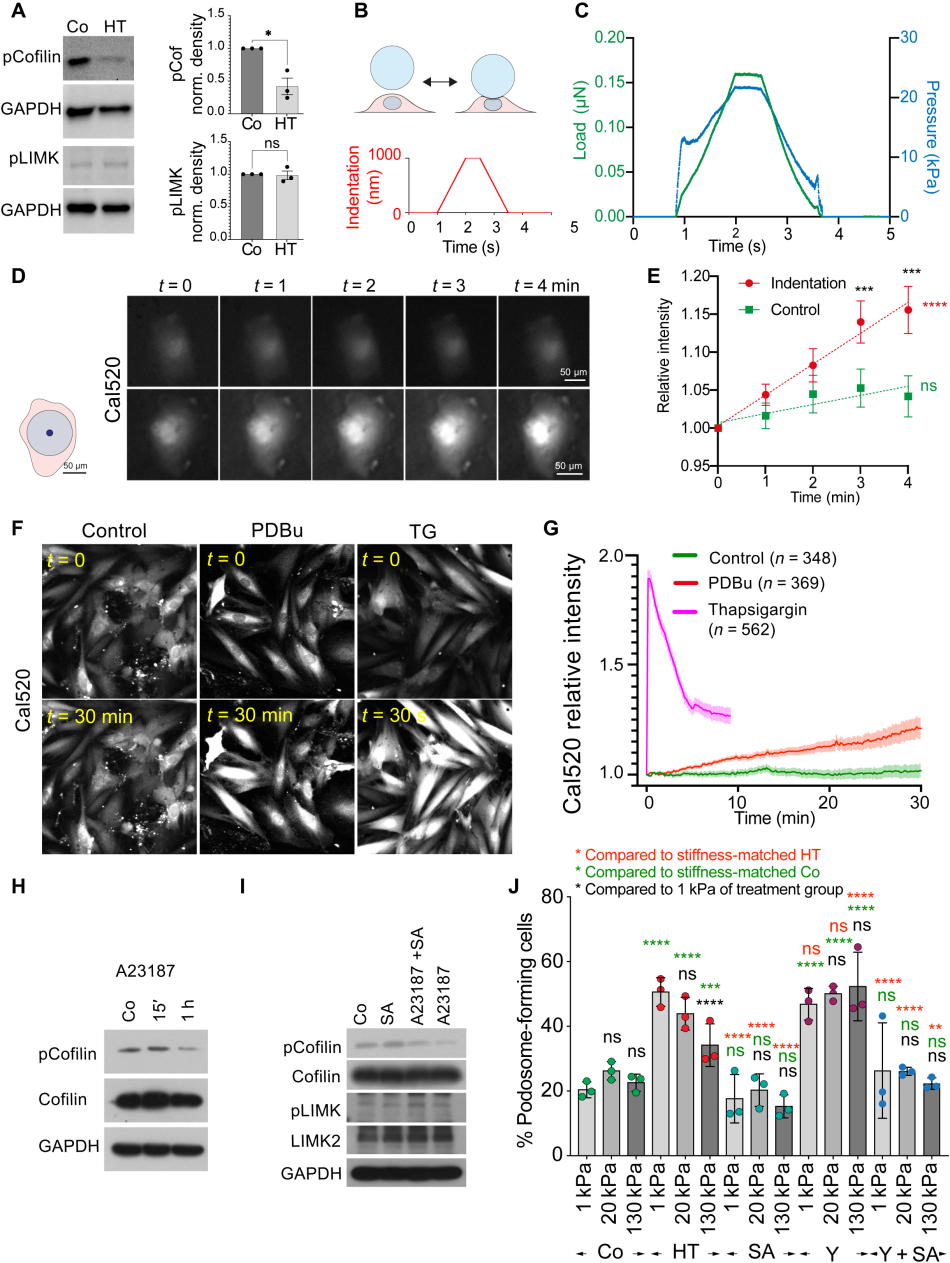
Pressure and PDBu stimulation induce calcium influx to regulate slingshot-dependent cofilin phosphorylation. (**A**) Hydrodynamic pressure stimulation reduces cofilin, but not LIMK phosphorylation. (**B**) Schematic of pressure application with the nanoindenter (top) and indentation settings (bottom). (**C**) Example traces of corresponding load and pressure. (**D** and **E**) Indentation leads to a consistent increase in Cal520 intensity. Schematic indicates size of bead (in blue) and contact area at 1-μm indentation (in dark blue) in comparison to cell area (in pink). (E) Quantification of *n* = 15 indented and 10 control cells (located nearby the indented cells) from three independent experiments. Error bars: SD. (**F** and **G**) PDBu treatment leads to a prolonged increase in Cal520 intensity, while TG induces a fast release of calcium. (G) Quantification of *n* = 348, 369, and 562 cells for control, PDBu, and TG treatment, respectively. (**H**) A23187 reduces cofilin phosphorylation after 1 hour. (**I**) SSH inhibition by sennoside A (SA) increases cofilin phosphorylation after PDBu treatment, and the reduction in pCofilin after A23187 is counteracted by simultaneous SSH treatment. (**J**) SSH inhibition [either alone or together with Y27632 (Y)] blocks podosome formation after hydrodynamic pressure stimulation on all stiffnesses, while ROCK inhibition enhances podosome formation after hydrodynamic pressure stimulation on all stiffnesses to a level otherwise seen on compliant surfaces only. *P < 0.0332; **P < 0.0021; ***P < 0.0002; ****P < 0.0001; *P* values from ANOVA and Bonferroni test for multiple comparisons or *t* test (E). (E) Black asterisks represent comparison for single time points and different conditions, and green and red asterisks represent *P* values from linear regression and test for deviation from zero. (J) Green asterisks represent comparison to the controls of same stiffness group, green asterisks represent comparison to the HT condition of same stiffness group, and black asterisks represent comparison to 1 kPa of same treatment group.

**Fig. 8.**
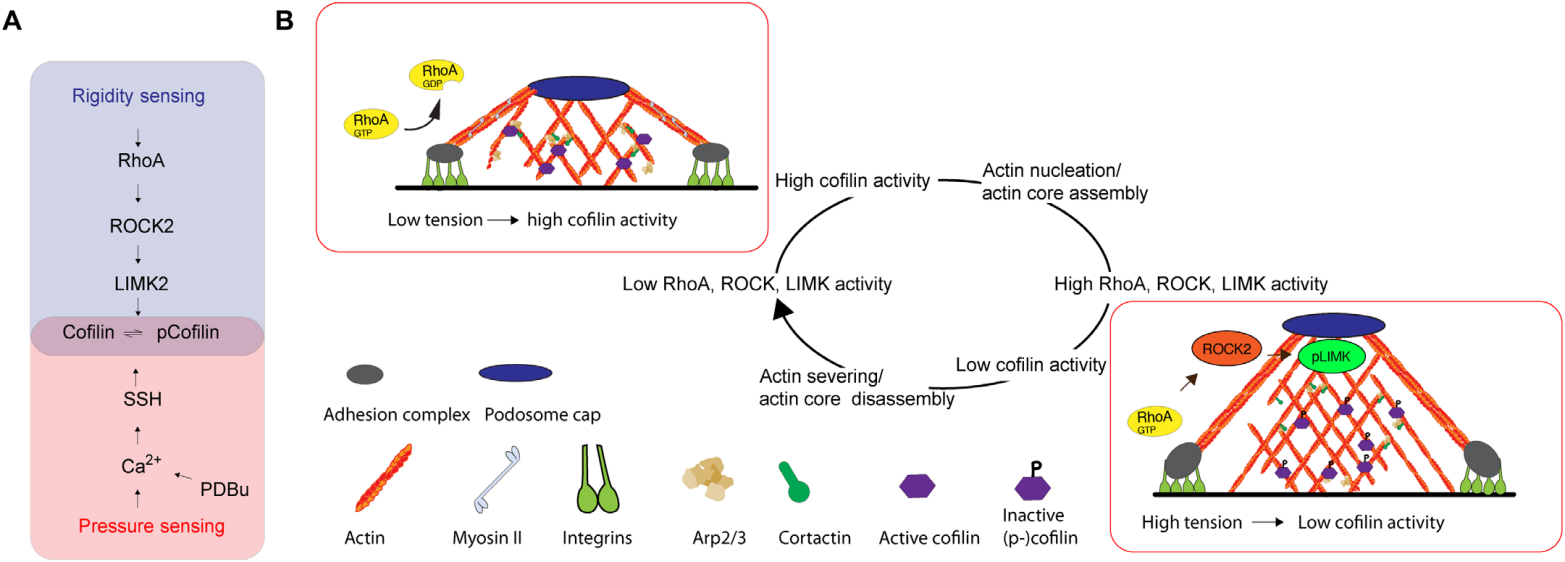
Schematic summarizing the pressure- and stiffness-dependent formation of podosomes in VSMCs. (**A**) Pressure and PDBu signal through Ca^2+^ and SSH, while rigidity sensing acts through RhoA, ROCK2, and LIMK2 to modulate cofilin phosphorylation. (**B**) Because cofilin switches between nucleation and severing depending on the concentration (*49*), fluctuations in cofilin activity result in a cyclic nature of podosome formation: High cofilin activity leads to actin nucleation and actin core assembly of podosomes, which eventually invokes RhoA activation at the podosome ring (through a currently unknown mechanism), thereby increasing ROCK and LIMK activity. This, in turn, reduces the cofilin activity, leading to actin severing and actin core disassembly and a reduction in RhoA activity.

While the RhoA-ROCK-LIMK pathway regulated the stiffness dependence of cofilin activity and podosome formation, we also found that the level of cofilin phosphorylation was decreased after cyclic HT pressure stimulation (Fig. 7A). However, the phosphorylation of LIMK remained unchanged, suggesting an alternative pressure-dependent mechanism to regulate cofilin phosphorylation. Mechanosensitive channels have received wide attention as regulators of cellular behavior since their discovery in the 1980s and especially the cardiovascular system is highly dependent on the regulation through mechanosensitive ion fluxes (*11*, *55*, *56*). In particular, elevated Ca^2+^ levels have been shown to promote the phosphatase activity of slingshot, which dephosphorylates cofilin (*57*). Besides, PKC (activated through PDBu treatment) not only is modulated by Ca^2+^ but also affects Ca^2+^ handling (*58*). Therefore, we hypothesized that hydrodynamic pressure could lead to an increase in baseline Ca^2+^ levels to induce podosome formation. When VSMCs were subject to a single indentation with a maximum pressure of 24 ± 4.4 kPa (180 ± 33 mmHg; Fig. 7, B to E), we found a consistent increase in intracellular Ca^2+^ levels, reported by the calcium indicator Cal520. Similar to pressure stimulation, PDBu treatment also resulted in a consistent increase in intracellular Ca^2+^ levels (Fig. 7, F and G). Notably, both the pressure and the PDBu stimulation only led to a slow increase, compared to the treatment with thapsigargin (TG), suggesting that both were inducing the influx of extracellular Ca^2+^ rather than the release from the intracellular calcium stores (Fig. 7, F and G). To examine the impact of Ca^2+^ influx on the cofilin phosphorylation, we found that the treatment with the divalent cation ionophore A23187 resulted in the decrease of cofilin phosphorylation, similar to PDBu (Fig. 7H). When the phosphatase activity of slingshot was inhibited by sennoside A (*59*), the level of cofilin phosphorylation increased (Fig. 7I). In addition, the effect of A23187 on phospho-cofilin was counteracted by simultaneous treatment with sennoside A, whereas phosphorylation of LIMK was unaffected by SSH inhibition, either with or without A23187 (Fig. 7I). Consistently, podosome formation (after HT pressure stimulation) was blocked after slingshot inhibition (either alone or in the presence of Y27632), while VSMC efficiently formed podosomes on all stiffnesses after ROCK inhibition with Y27632 (Fig. 7J). Together, these results suggest that hydrodynamic pressure and PDBu treatment are both acting on cofilin phosphorylation through calcium and slingshot phosphatase to induce podosome formation, while stiffness sensing is modulating cofilin phosphorylation through the RhoA-ROCK-LIMK signaling axis (Fig. 8). Because VSMCs lose the link between rigidity sensing and podosome formation after SSH inhibition (likely due to loss of intrinsic podosomal rigidity sensing—see also Figs. 3, C to E, and 5, E to H), we conclude that both signals are acting together to lead to a change in VSMC phenotype, podosome formation, and extracellular matrix remodeling and ultimately contribute to the progression of atherosclerotic diseases.

## DISCUSSION

Atherosclerosis is a severe cardiovascular disease. Mechanical stimuli have been strongly implicated in both disease onset and progression. The role of wall stress on ECs is well documented and determines atheroprone and atheroprotective regions (*6*). In addition, aging (and associated changes to the mechanical properties of the arterial wall) and HT are major risk factors (*1*–*5*, *60*). Especially, the effect of blood pressure on the arterial wall changes during aging and in disease. The enhanced macroscale stiffness leads to a reduced distension but increased radial stress and compression of the intima layer (*23*, *35*, *37*, *46*). These mechanical stimuli also affect the residing VSMCs, which are critically involved in the early stages of atherosclerosis (*13*). However, the effects of the different overlapping physiological or pathological mechanical stimuli on intimal VSMCs are still unclear.

Here, we sought to investigate the role of the individual mechanical stimuli through hydrodynamic pressure stimulation of cells on surfaces with defined stiffness, as well as separate cell stretching experiments. This way, we can show that two mechanical stimuli, when combined, compliance (mimicking the intima or pre-atherosclerotic lesions), and hypertensive pressure lead to a phenotypic switch of VSMCs. This effect can be observed for the A7r5 VSMC line, as well as primary bovine and human VSMCs. In all cases, the matrix stiffness affects the cell morphology and cytoskeleton (Fig. 1 and fig. S1). This seemingly makes the cells more susceptible to further phenotypic changes due to hypertensive pressure. Hence, maximal phenotypic switching is strongly dependent on the combination of pressure treatment (or chemical stimulation) and compliant environment. The result is enhanced podosome formation, remodeling of the extracellular matrix, and a further reduction of the extracellular matrix stiffness (Fig. 6, A to C). This is further confirmed by our proteomic data, which only picked up significantly different protein levels between pressure-treated and control samples, when these were seeded on compliant surfaces. Consistent with the observed phenotypic change, we detected a range of molecules that were previously associated with podosome formation and/or atherosclerosis among the differential regulated proteins. The implication of this finding is significant for the processes seen during early atherosclerosis, where the combination of compliance with hypertensive pressure will enhance the phenotypic switch and accelerate matrix remodeling, including enhanced expression of proteoglycans in the vessel wall (Fig. 6D) and atherosclerotic progression in a downward spiral.

It is important to mention that our findings are not contrasting the importance of endothelial mechanosensing in onset and progression of atherosclerosis (*6*, *12*). Rather, our data suggest that chemical signals sent from the endothelium can initiate the phenotypic switch in VSMCs through various chemical signaling pathways, including platelet-derived growth factor (PDGF) and Wnt pathways that activate PKC (*12*, *61*, *62*) and thus will affect calcium influx (*58*), cofilin phosphorylation (*57*), and podosome formation. Similarly, HT and atherosclerosis have been both linked to up-regulations of vasoconstrictor endothelin-1 (*63*) and angiotensin II (*64*), which likewise lead to increased production of diacylglycerol and downstream activation of PKC (*65*, *66*). This shows that mechanical and chemical signals are not mutually exclusive but rather act together to speed up the disease progression, whereby the downstream formation of podosomes contributes to the remodeling of the pre-atherosclerotic lesions.

Mechanosensitive behavior of podosomes was previously studied in monocytic cells, with differing outcomes. One study using dendritic cells on a fibrinogen matrix found an increase in podosome formation with increasing stiffness, while another study using macrophages found a higher degree of podosome formation on compliant fibronectin-coated hydrogels (*67*, *68*). Similar to the latter study, we find a larger degree of podosome formation and dynamicity on soft fibronectin, gelatin, or collagen-coated (not shown) surfaces. We also detect a fluctuating appearance of podosomes at the same locations, whereby the oscillation period matches well with force oscillations that were previously observed in podosomal force studies (*67*, *69*–*71*).

A slow oscillating component was previously reported in the range of 7 ± 4 min, which matched the actin assembly period at the podosome core (*70*, *71*). Fluctuating actin assembly, in turn, is consistent with the dual activity of cofilin as actin nucleator and severing factor, depending on its (active) concentration (*49*), regulated downstream of RhoA signaling. As the substrate stiffness orchestrates RhoA activity, the downstream pathway of ROCK and LIMK will affect cofilin phosphorylation and the overall propensity of cells to initiate podosome formation.

The level of RhoA-GTP is decreased on compliant surfaces, leading to the cofilin-mediated actin nucleation and a larger fraction of cells forming podosomes after pressure or chemical stimulation (*51*, *52*). Moreover, once cells start forming podosomes, the regulation of RhoA-GTP level can locally form a feedback loop to dynamically modulate the cofilin activity and myosin-dependent oscillation. The increase of RhoA-GTP level during the assembly phase of podosome can reduce the activity of cofilin in actin nucleation and lead to the subsequent podosome disassembly.

Together, this suggests that stress- or strain-dependent effects might lead to the activation of RhoA and point toward a role for a negative regulator of RhoA activity in the podosome adhesion ring. At this moment, we can only speculate about the upstream mechanosensor. Potential molecules include ARAP3, a dual guanosine triphosphatase (GTPase)–activating protein (GAP) for RhoA and Arf, which is dynamically recruited to the podosome in a focal adhesion kinase (FAK)/phosphatidylinositol 3-kinase (PI3K)–dependent manner (*52*). Our quantitative proteomics analysis found that ARAP3 is strongly up-regulated after pressure stimulation, especially on soft surfaces, consistent with a higher dynamicity of podosomes. Other BAR domain containing RhoA GAPs, such as srGAP1, RICH1, and GMIP, might sense the change of membrane curvature during the podosome formation (which we detect even on glass coverslips, where podosomes appeared to lift the membrane upward in their vicinity; fig. S9) (*72*) and regulate local RhoA activity (*73*, *74*). However, future experiments are needed to untangle the stiffness-dependent regulation of RhoA activation at the adhesion ring. The current study primarily used two-dimensional (2D) cell culture models, which enabled the detailed analysis of VSMC mechanosensing pathways. However, arteries are 3D structures and cells in the arterial wall are surrounded by extracellular matrix and exposed to forces in all three dimensions. While we used in vivo data to validate our findings, future work is needed to further disseminate the regulation of VSMC mechanosensing in 3D models.

Overall, our findings suggest a regulation of VSMC phenotypic switching through combined mechanical signaling through hemodynamic pressure and extracellular matrix stiffness, which could be targeted to mitigate early atherosclerotic processes and abate disease progression.

## MATERIALS AND METHODS

### Antibodies and reagents

Primary antibodies included vinculin [Sigma-Aldrich, V9131, 1:200 for immunofluorescence (IF)], cortactin (Sigma-Aldrich, 4F11, 1:100 for IF), phospho-cofilin (Ser^3^) [Cell Signaling Technology (CST), 3311, 1:3000 for Western blot (WB)], cofilin (D3F9, CST, 5175, 1:5000 for WB and 1:100 for IF), phospho-cortactin (Tyr^421^) (Thermo Fisher Scientific, 44854G, 1:1000 for WB), phospho-LIMK1 (Thr^508^)/LIMK2 (Thr^505^) (CST, 3841, 1:1000 for WB), LIMK1 (CST, #3842 or BD Biosciences, clone #42, 1:1000 for WB), LIMK2 (Abcam, ab45165 or CST, #8C11, 1:1000 for WB and 1:100 for IF), PAK1/2/3 (CST, 2604, 1:1000 for WB), phospho-PAK1/2/3 (Thr^402^) (Thermo Fisher Scientific, PA1-4636, 1:1000 for WB), phospho-PAK4 (Ser^474^)/PAK5 (Ser^602^)/PAK6 (Ser^560^) (CST, 3241, 1:1000 for WB), PAK4 (CST, 62690 or 3242, 1:1000 for WB), ROCK2 (D1B1, CST, 9029, 1:1000 for WB), ROCK1 (C8F7, CST, 4035, 1:1000 for WB), pan-actin (Merck, #MAB1501), chondroitin sulfate (BD Biosciences, #554275), β-tubulin (CST, 2146, 1:3000 for WB), and glyceraldehyde-3-phosphate dehydrogenase (GAPDH) (Thermo Fisher Scientific, AM4300 or Abcam ab9485, 1:10,000 for WB). Alexa Fluor 488 phalloidin and Alexa Fluor 568 phalloidin were from Invitrogen.

The following reagents were used at the specified concentrations after 24-hour serum starvation: PDBu (1 μM; Tocris, #4153), TG [100 nM; Santa Cruz Biotechnology (SCBT), sc-24017], A23187 (1 μM; SCBT, sc-3591), sennoside A (10 μM; Sigma-Aldrich, 68909-5MG-F or Selleck, #S4033), Y27632 (10 to 20 μM; Cambridge Bioscience or Selleck, #S1049), H1152 (10 μM; Tocris, #2414), KD025 (10 μM; Selleck, #S7936), ML141 (10 μM; Selleck, #S7686), and A23187 (Selleck, #S7778).

### Plasmids

Cofilin wild type (WT), cofilin S3A, and cofilin S3E-pmiRFP were created by subcloning the respective cofilin constructs from pEGFP-N1 human cofilin plasmids using Hind III–HF and Xma I restriction sites. pEGFP-N1 human cofilin WT, cofilin S3A, and cofilin S3E were gifts from J. Bamburg (Addgene, plasmid nos. 50859, 50860, and 50861). pmiRFP703-N1 and pLifeAct-miRFP703 were gifts from V. Verkhusha (Addgene, plasmid nos. 79988 and 79993). mEos2-Actin-7 was a gift from M. Davidson (Addgene, plasmid no. 57339). GFP-ROCK2 was a gift from A. Yap (Addgene, plasmid no. 101296). pLKO.1-TRC cloning vector was a gift from D. Root (Addgene, plasmid no. 10878). Tractin-tomato and tractin-GFP were gifts from M. Schell. The RhoA-FRET biosensor was a gift from O. Pertz. mTFP-TRAF-Venus and mTFP-5AA-Venus were gifts from N. Borghi. Vinculin-mTFP and vinculin-Venus were gifts from C. Grashoff.

### Short hairpin RNA

The following sequences, inserted into an empty pLKO.1-TRC vector, were used for short hairpin RNA (shRNA) knockdowns: shScramble, 5′-CCTAAGGTTAAGTCGCCCTCG-3′; shROCK1#1, 5′-GGTTTATGCTATGAAGCTTCT-3′; shROCK1#2, 5′-GCATTTGCCAATAGTCCTTGG-3′; shROCK1#3, 5′-GCCGACTTTGGTACTTGTATG-3′; shROCK1#4, 5′-GCACCAGTTGTGCCTGATTTA-3′; shROCK2#1, 5′-TGCAAAGTTTATTATGATATA-3′; shROCK2#2, 5′-AACGTGGAAAGCCTGCTGGAT-3′; shROCK2#3, 5′-GCAGAAAGTTCCAAACAGA-3′; shROCK2#4, 5′-GTAGAAACCTTCCCAATTC-3′; shLIMK1#1, 5′-CCTCCATTCGATGAACATCAT-3′; shLIMK1#2, 5′-AAGACTTGCGTAGCCTTAAGA-3′; shLIMK1#3, 5′-AATGCAGACCCTGACTATCTG-3′; shLIMK2#1, 5′-AATGGCAAGAGCTACGATGAG-3′; shLIMK2#2, 5′-AACAACCGAAATGCCATCCAC-3′; shLIMK2#3, 5′-GCCATCAAGGTGACTCACAAA-3′; all shRNA oligos were from Integrated DNA Technologies. Cells were transfected with shRNAs for 96 hours before the experiments.

### Cell culture, immunostaining, and microscopy

Primary VSMCs were obtained from explants of bovine aorta and from human aortic tissue from two healthy female donors aged 35 and 38 years, as previously described (*75*). Bovine VSMCs were used for the experiments at passages 6 to 8. Human VSMCs were used at passages 5 to 8 (cell line 1: WT 03:38F:9A) and passages 10 to 12 (cell line 2: WT 04-35F-11A). Primary cells were cultured in Dulbecco’s modified Eagle’s medium (DMEM) containing 20% fetal bovine serum (FBS). A7r5 rat arterial VSMCs were cultured in DMEM with 5% FBS, 1% GlutaMAX, and 1% penicillin/streptomycin. Cells were transfected using Lipofectamine LTX with Plus Reagent, following the manufacturer’s instructions. For immunostaining, cells were fixed with 4% paraformaldehyde for 10 min, permeabilized with 0.2% Triton X-100 in phosphate-buffered saline (PBS) for 5 min, blocked with 5% bovine serum albumin (BSA) in PBS for 1 hour, and stained in antibody solutions in immunostaining buffer (20 mM tris, 155 mM NaCl, 2 mM EGTA, 2 mM MgCl_2_, 1% BSA at pH 7.4). Cells were washed three times for 10 min with PBS after each step and mounted in MOWIOL 4-88 [0.1 g/ml in glycerol/water/tris (0.2 M, pH8.0) at a ratio of 1:1:2] containing a final concentration of 4% *n*-propyl gallate. Live-cell imaging was performed on an inverted Nikon Eclipse Ti-E microscope with a Nikon DS-Qi2 sCMOS camera (podosome lifetime imaging) or a Nikon Ti2 SoRa spinning disc microscope with two Photometrics Prime BSI cameras for simultaneous imaging (FRET, calcium imaging, used in confocal mode), both equipped with environmental chamber with temperature and CO_2_ control. Podosome IF imaging was performed on a Nikon Ti2 SoRa spinning disc microscope with a 60× 1.49–numerical aperture objective in SoRa 4× magnification mode. Imaging of cells after pressure stimulation was done on a Leica DMI8 epifluorescence microscope with a Leica DFC9000 GT camera.

Fluorescence loss after photoconversion experiments was performed with a PerkinElmer UltraView VOX spinning disc microscope. The photoconvertible mEos2-actin was excited by an instant pulse of 405-nm ultraviolet laser at cofilin-positive podosomes. The decay phase of the photoconverted red mEos2-actin was then imaged at the rate of 1 s per frame.

### Animal model and tissue sections

Complete ligation of the left common carotid artery (LCCA) was performed with 8-week-old male C57BL/6 mice. After harvesting 28 days after ligation, the proximal and distal 2 to 3 mm of LCCA were discarded and the remaining portion (4 to 5 mm) was embedded in optimal cutting temperature compound for frozen sectioning on a cryostat microtome (Leica Biosystems). Frozen sections (10 μm thick) were prepared and subjected to IF staining analyses with the indicated antibodies.

For this, frozen sections were thawed at room temperature for 15 to 20 min and rehydrated twice with PBS for 5 min. Tissues were blocked for 30 min with 5% horse serum diluted in PBS and incubated with primary antibodies (1:100) at 4°C overnight. The next day, sections were washed with PBS for 5 min three times and incubated with secondary antibodies (1:200) for 60 min at RT. Tissues were washed three times with PBS, incubated for further 15 min with 4′,6-diamidino-2-phenylindole (DAPI) (1:1000), washed again with PBS, and mounted using ProLong Gold mounting media (Thermo Fisher Scientific). Z-stacks of the 10-μm-thick sections were taken on a Nikon Ti2 SoRa spinning disc microscope with a 60× oil objective (unless otherwise specified). Maximum intensity projections were calculated, and staining intensities of cells were measured using ImageJ. The indicated number of cells from three sections each from *n* = 3 to 5 animals was included in the analysis and plotted as mean per animal.

### PDMS substrates

Flat polydimethylsiloxane (PDMS) substrates were prepared as described previously (*76*). Briefly, Sylgard 184, Sylgard 527, or mixtures at the ratios of 1:5, 1:10, and 1:20 were spin-coated with a 150i spin processor (SPS) onto coverslips. Before spin coating, Sylgard 527 was precured at 70°C for 30 min with intermitted mixing to achieve a comparable viscosity to the Sylgard 184 mixture. The stiffness of mixtures was measured by rheology as described previously (*38*). PDMS substrates were coated with fibronectin or DQ gelatin for matrix degradation experiments.

### Pressure stimulation

Hydrodynamic pressure stimulation was performed in a MechanoCulture TR stimulator, placed inside a tissue culture incubator. The stimulator was modified for low-pressure stimulation in the range of (human) normal (NBP) and hypertensive blood pressure (HT) and perfusion ports to supply presaturated cell culture medium. Cells were stimulated with a sinusoid profile (stretch: 0.5 s, duration: 1 s, hold: 0 s, recovery: 0.5 s) and alternating between set pressures of 16 kPa (peak load) and 8 kPa (pre-load) to reach a measured pressure profile of 120/60 mmHg for NBP stimulation and between 26 and 16 kPa for a measured pressure profile of 180/120 mmHg for hypertensive pressure stimulation. Cyclic pressure stimulations were performed for 12 hours, and static pressure stimulations (at peak pressure setting) were performed for 30 min. Control cells were placed inside the stimulator without applying pressure.

### Cell stretching

Cell stretching experiments were performed using a FlexCell-4000 setup. Cells were seeded onto BioFlex Collagen I plates at ~50% confluence for biaxial strain stimulation the following day. Cells were stretched using a sinusoid profile cycling between 0 and 5% or 0 and 10% strain. Unstretched control cells were seeded onto unstretched membranes in parallel, retained in the tissue culture incubator, and then fixed simultaneously once the experiment had finished.

### Nanoindentation

Nanoindentation experiments were performed using an Optics11 Chiaro nanoindenter attached to a Leica DMI-8 microscope. Cell measurements were performed above the nucleus with an *R* = 50 μm, *k* = 0.5 N/m probe (suitable for a stiffness range between approximately 0.5 and 80 kPa). The Hertzian contact model was used to fit the data. The contact point was identified from a contact point fit of the data to 20% of the maximal load and used to subsequently fit the Young’s modulus using an indentation depth of 1 μm.

For tissue sections, measurements were performed with an *R* = 50 μm, *k* = 0.5 N/m probe, and 500-nm indentations in the matrix scan mode. Images of the section with the probe in contact at the start and end position were taken for alignment of the measurements with bright-field image. Only measurements overlapping with the arterial wall were included in the analysis.

### Ca^2+^ measurements

For calcium measurements, cells were plated on fibronectin-coated PDMS coverslips and loaded with Cal520 AM (Abcam, ab171868) according to the manufacturer’s instructions.

Cells were then treated with 1 μM PDBu or 1 μM TG and imaged on a Nikon Ti2 SoRa spinning disc microscope in confocal mode (50-μm pinhole) with a Plan Fluor 10× air objective. Untreated cells were used as a control. To measure the calcium response after pressure stimulation, controlled force was applied onto cells using a Chiaro nanoindenter with an *R* = 49 μm and *k* = 0.46 N/m probe, using an indentation depth of 1 μm (equaling a contact radius of 7 μm) with a maximum pressure of 24 ± 4.4 kPa.

### Gelatin degradation

DQ gelatin is a fluorogenic substrate that emits green light once degraded by matrix metalloproteinases. To build a thin layer of gelatin on PDMS, 25 mm coverslips precoated with PDMS were treated with 18 W air plasma for 1 min and then immediately coated with 0.1% DQ gelatin solution (DQ gelatin from pig skin, fluorescein conjugate, D12054) at room temperature for 10 min in a light-protected sterilized hood. Excess gelatin solution was removed using PBS. A7r5 cells were seeded at ~50% confluence 1 day before the experiment (PDBu treatment of pressure stimulation). For quantification of DQ gelatin degradation, a Fourier bandpass filter was applied to increase the signal-to-noise ratio of the DQ gelatin channel. The filtered images were combined with the actin and DAPI channels and analyzed using a cell profiler pipeline. Briefly, DQ gelatin–positive areas were detected as primary objects, masked by the previously identified cell areas, and then combined and measured as sum of the degraded area per cell.

### Image analysis

Image segmentation was performed using CellProfiler, ImageJ (OrientationJ), and MATLAB as described previously (*77*). Briefly, OrientationJ produces a weighted histogram for pixels per orientation. The weight is the coherency, which is defined through the ratio of difference and sum of the tensor eigenvalues and is bounded between 0 and 1, with 1 representing highly oriented structures (*39*). In addition, we added actin segmentation to the CellProfiler pipeline and trained an Ilastik pixel classification to detect actin and cortactin dots. All measurements were combined, and dimensionality reduction was performed in MATLAB using the t-SNE algorithm. Cluster borders were drawn manually, and populations from each separate repeat were calculated. Additional cell morphological analysis was performed with the visually aided morphophenotyping image recognition VAMPIRE software (*40*).

For analysis of fluorescence loss after photoconversion, mEos2-actin intensity decay profiles of each podosome were measured by ImageJ and fitted into a one-phase decay curve. FRET analysis was performed in ImageJ. First donor and acceptor bleed through were determined using vinculin-mTFP and vinculin-Venus constructs using the PixFRET plugin. Donor bleed through was best fit with a constant value, and acceptor bleed through was determined to be negligible. Movies were processed using ImageJ macro by subtracting the background from all channels, calculating the bleed through corrected donor image, subtracting the bleed through corrected donor image from the FRET image, and then normalizing this by the donor intensity.

### RhoA-GTP pull down

A7r5 cells were seeded on the newly prepared fibronectin-coated PDMS substrate with the defined stiffness for 48 hours and then treated with 1 μM PDBu for 2 hours. To detect the RhoA-GTP level, cell lysates were prepared according to the manufacturer’s instructions (RhoA Pull-Down Activation Assay Biochem Kit, Cytoskeleton Inc., BK036). Specifically, lysates with 300 μg of protein were incubated with the Rhotekin-RBD–containing agarose bead at 4°C on a rotator. Total RhoA in the cell lysate and activated form of Rho-GTP in the agarose bead were detected by WB.

### Quantitative proteomic analysis

Quantitative proteomic analysis was performed at the Proteomics Facility Denmark Hill, King’s College London. Samples were loaded onto 1D SDS gel for in-gel reduction, alkylation, and digestion with trypsin, before subsequent analysis by mass spectrometry (MS). Digested peptides were labeled with TMT16plex tags, according to the protocol provided by the manufacturer.

Each TMT set sample was resuspended in resuspension buffer (2% acetonitrile in 0.05% formic acid) to be analyzed by liquid chromatography (LC)–MS/MS with triple injections. Chromatographic separation was performed using an Ultimate 3000 NanoLC system (Thermo Fisher Scientific, UK). Peptides were resolved by reversed-phase chromatography on a 75 μm–by–50 cm C18 column using a three-step gradient of water in 0.1% formic acid (A) and 80% acetonitrile in 0.1% formic acid (B). The gradient was delivered to elute the peptides at a flow rate of 250 nl/min over 100 min.

The eluate was ionized by electrospray ionization using Orbitrap Fusion Lumos (Thermo Fisher Scientific, UK) operating under Xcalibur v4.1. The instrument was programmed to acquire using a “Synchronous Precursor Selection with MultinotchMS” method (SPS) for accurate and sensitive quantitation based on isobaric TMT tags.

Raw MS data were processed into peak list files using Proteome Discoverer (Thermo Fisher Scientific, v2.5). Processed data were then searched using Sequest search engine embedded in PD 2.5, against the current version of the reviewed Swiss-Prot Rat database downloaded from UniProt.

The LC-MS/MS analysis successfully identified more than 10,000 protein groups containing at least one peptide identified across all samples with a peptide cutoff threshold of false discovery rate (FDR) 0.05. After peptide sequence identification, default thresholds (Co-Isolation threshold: 50; Average S/N threshold: 10; SPS Mass Matches threshold: 65) were applied during the reporter quantification to remove redundant peptide spectrum matches (PSMs). Proteins and peptides were then quantified on the basis of reporter ion ratios (signal to noise) of nonredundant PSMs. Among more than 10,000 identified protein groups, 1026 protein groups were quantified. Because the peptide cutoff was set up as FDR 0.05, a further filtering with an Xcorr score greater or equal to 1.5 and valid present over 70% across replicates within each condition was applied, aiming to remove poor identification and quantification. As a result, 1026 quantified protein groups were used for downstream differential analysis (tables S1 to S5). Cluster analysis and pairwise differential analysis were carried out using the MATLAB clustergram using the correlation distance metering and mavolcanoplot functions.

### Statistical analysis

Datasets were tested for normal distribution using the Shapiro-Wilk test. All statistic tests were performed with GraphPad Prism using either *t* tests for two conditions or analysis of variance (ANOVA) and correction for multiple comparisons.

## Notes

View/request a protocol for this paper from *Bio-protocol*.
