# Supplementary material for: Pressure and stiffness sensing together regulate vascular smooth muscle cell phenotype switching
Source: Sci Adv. 2022 Apr 15;8(15):eabm3471. doi: 10.1126/sciadv.abm3471 (PMC9012473; doi:10.1126/sciadv.abm3471)
Supplement: Supplementary file 1 — Figs. S1 to S9 [file sciadv.abm3471_sm.pdf]

Science Advances
AAAS

Supplementary Materials for

Pressure and stiffness sensing together regulate vascular smooth muscle cell phenotype switching

Pamela Swiatlowska, Brian Sit, Zhen Feng, Emilie Marhuenda, Ioannis Xanthis, Simona Zingaro, Matthew Ward, Xinmiao Zhou, Qingzhong Xiao, Cathy Shanahan, Gareth E. Jones, Cheng-han Yu*, Thomas Iskratsch*

*Corresponding author. Email: t.iskratsch@qmul.ac.uk (T.I.); chyu1@hku.hk (C.-h.Y.)

Published 15 April 2022, *Sci. Adv.* **8**, eabm3471 (2022)
DOI: 10.1126/sciadv.abm3471

The PDF file includes:

Figs. S1 to S9
Legends for tables S1 to S5
Legends for movies S1 to S3

Other Supplementary Material for this manuscript includes the following:

Tables S1 to S5
Movies S1 to S3

Supplementary Information

Supplementary Figures:

Supplementary Figure S1:

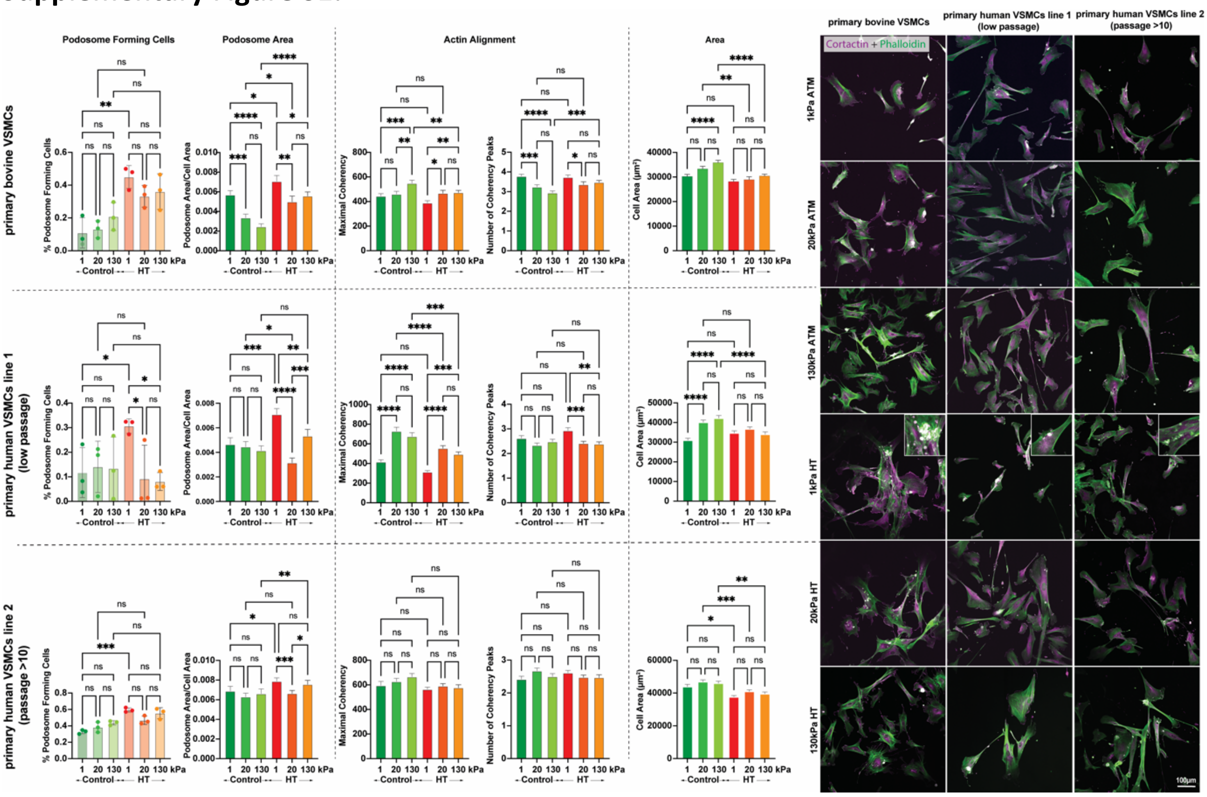

Supplementary Figure S1: Combined compliance and cyclic hypertensive pressure result in podosome formation and actin rearrangements also in primary bovine and human vascular smooth muscle cells. Cells were plated on PDMS coated coverslips with different stiffness (1,20,130kPa) and placed under cyclic hydrodynamic pressure mimicking hypertensive blood pressure (180/120mmHg). Cells were then fixed and stained with Phalloidin (green) and Cortactin (Magenta). F) HT treatment resulted in reduced actin organisation on 1kPa, cell area, as well as podosome forming cells and podosome area (pooled data from three separate experiments). Insets at 1kPa HT display some examples of podosome forming cells. Bovine VSMC were used for experiments at passages 6-8. Human VSMCs were used at passages 5-8 (cell line 1: WT 03:38F:9A) and 10-12 respectively (cell line 2: WT 04-35F-11A). $^{*}p < 0.0332$; $^{**}p < 0.0021$; $^{***}p < 0.0002$; $^{****}p < 0.0001$; ns, not significant; p-values from ANOVA test with Bonferroni correction for multiple comparisons.

Supplementary Figure S2:

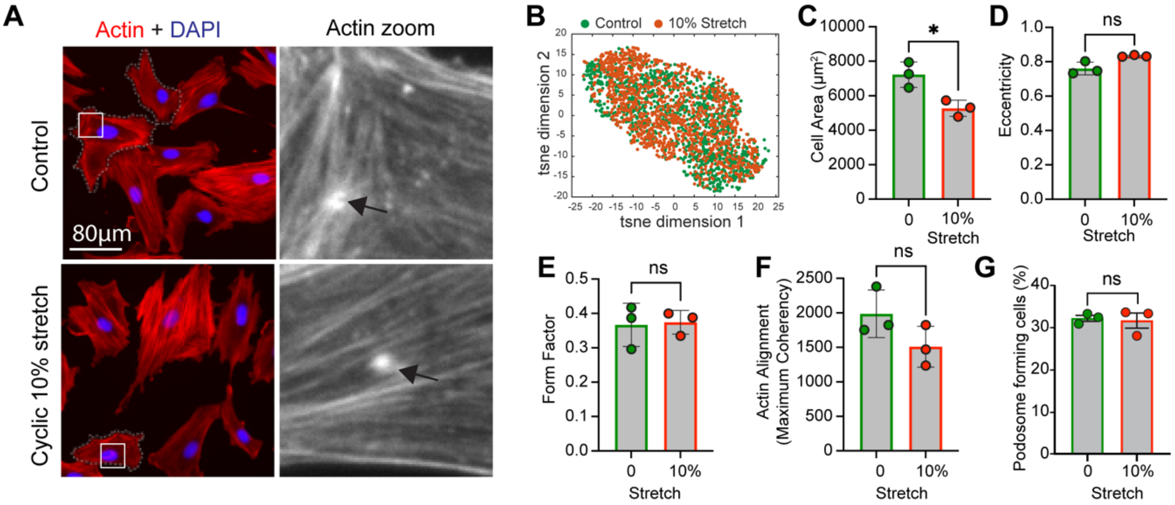

Supplementary Figure S2: Cell Stretch alone is not sufficient to induce podosome formation. A) A7r5 vascular smooth muscle cells were stained with phalloidin and DAPI. Right panel shows a zoom of the actin channel from the area indicated in the left panel. Arrows indicate podosomes, which were detected in ~30% of cells independent of stretch. B-G) Cells were analysed as reported for the pressure-stimulated cells in Fig 1. Both stretched and control cells cluster together (B). Cell area was slightly decreased (C), while no differences were observed for eccentricity (D), form factor (E), actin alignment (F) or podosome formation (G). C-G) data from 3 independent repeats, displayed as mean per repeat (I). *p < 0.0332; ns, not significant; p-values from unpaired t-test.

Supplementary Figure S3:

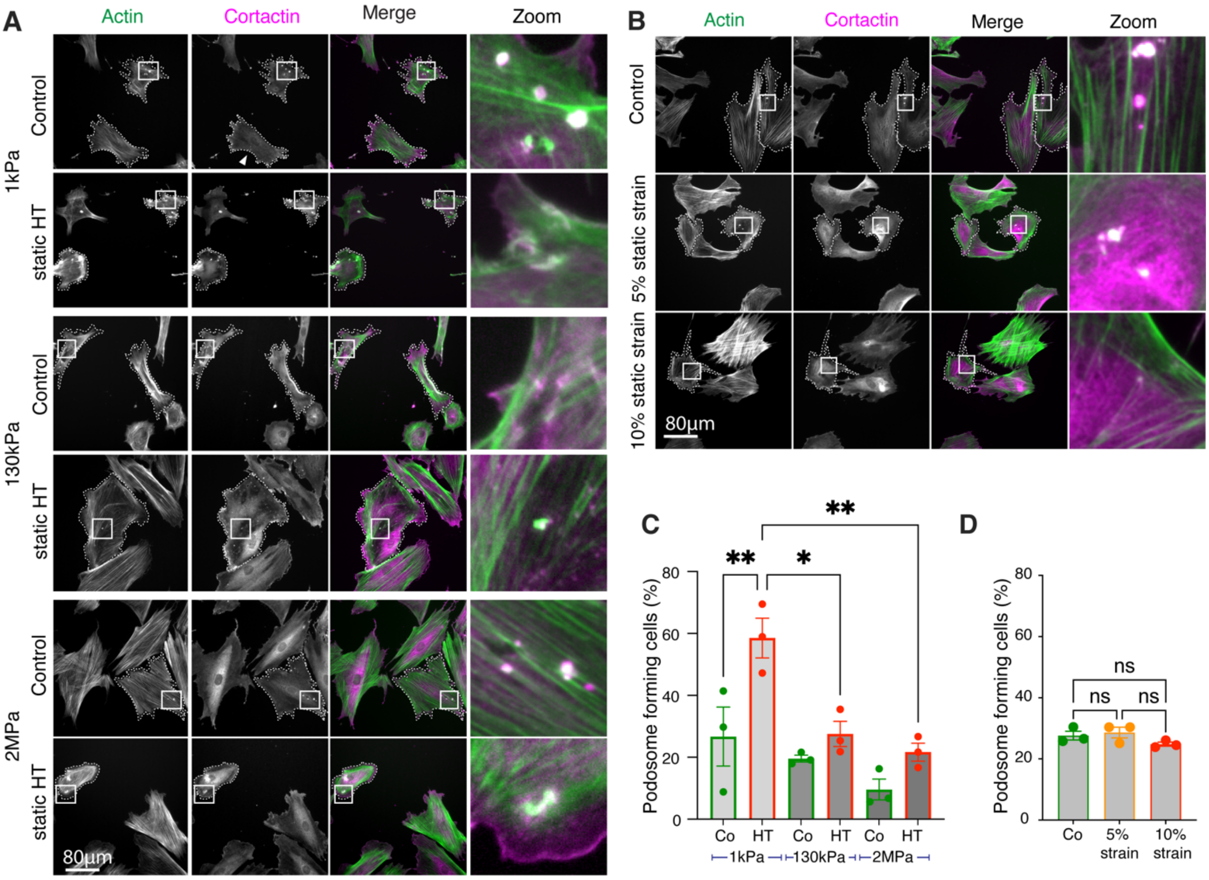

Supplementary Figure S3: VSMC form podosomes after 30minute static pressure, but not stretch. A-B) A7r5 VSMCs were subjected to 30min hydrostatic pressure at 24kPa or alternatively 5% or 10% static biaxial stretch and then stained for cortactin and F-actin. C,D) Quantification of the fraction of podosome forming cells from three independent experiments. *$p < 0.0332$; **$p < 0.0021$; ns, not significant; p-values from ANOVA test with Bonferroni correction for multiple comparisons. C: only significant differences are displayed.

Supplementary Figure S4:

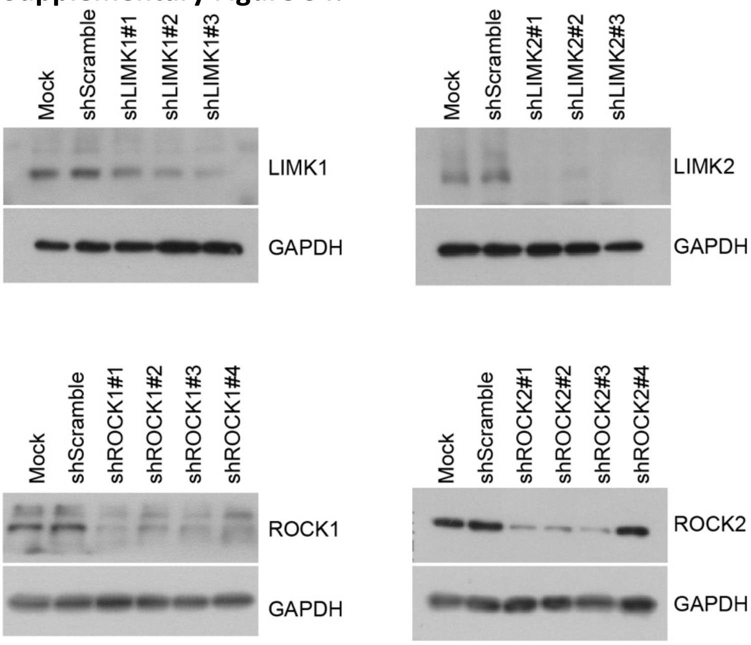

Supplementary Figure S4: Validation of shRNA knockdowns. Representative images from three independent repeats.

Supplementary Figure S5:

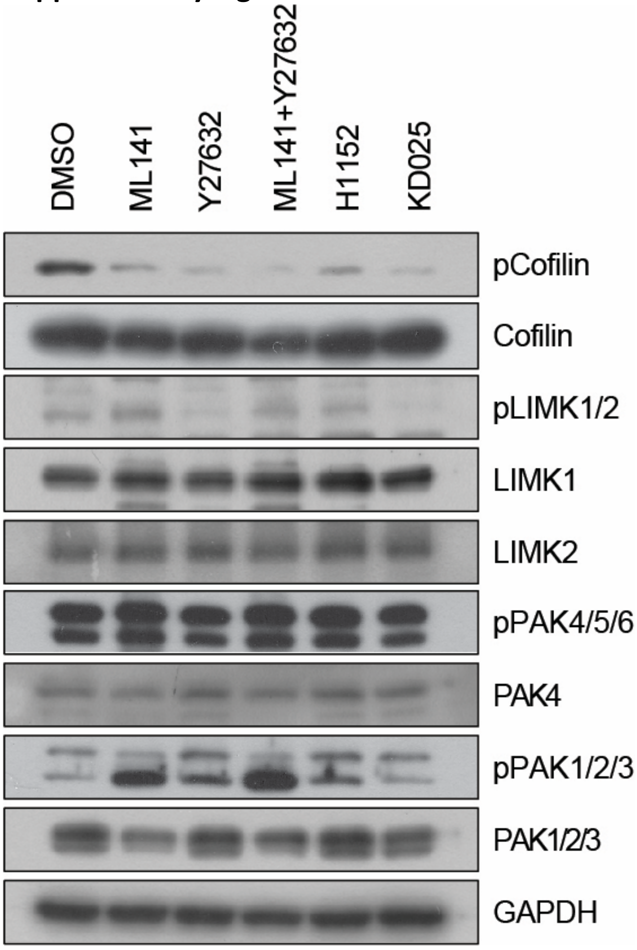

Supplementary Figure S5: Western blot analysis of effects of Cdc42 (ML141, 10µM), ROCK1/2 (Y27632, 20µM; H1152, 10µM) or ROCK2 (KD025, 10µM) inhibition. The inhibition of ROCK2 specifically supresses the phosphorylation of both LIMK and cofilin in A7r5 vascular smooth muscle cells. The inhibition of Cdc42 results in the dephosphorylation of cofilin and the phosphorylation of group 1 PAK (PAK1/2/3) while LIMK phosphorylation remains unchanged. The dual inhibition of ROCK and Cdc42 promotes further cofilin dephosphorylation. All inhibitors were applied to the cells for two hours.

Supplementary Figure S6:

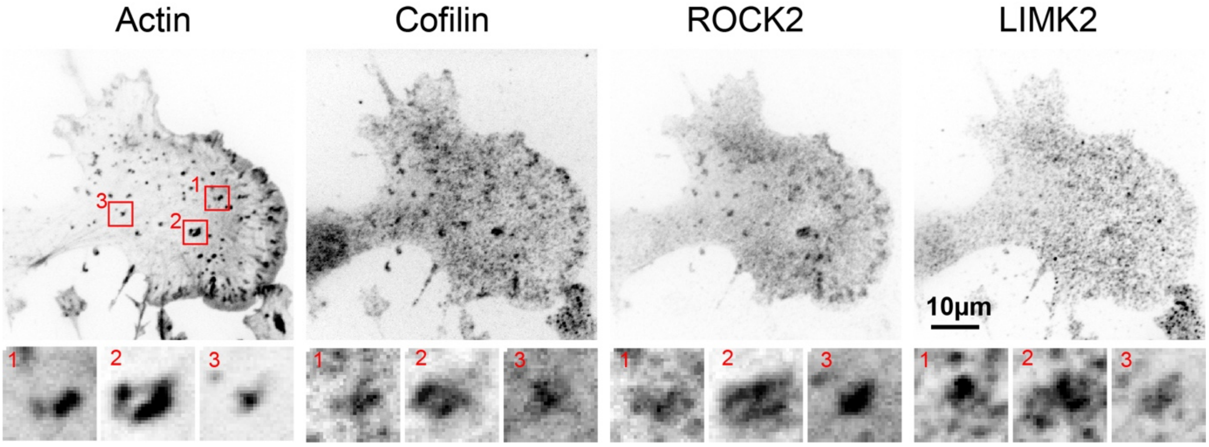

Supplementary Figure S6: ROCK2 and LIMK2 and cofilin localize to podosomes in A7r5 VSMCs. Bottom panel shows zoom of three regions marked in the actin channel.

Supplementary Figure S7:

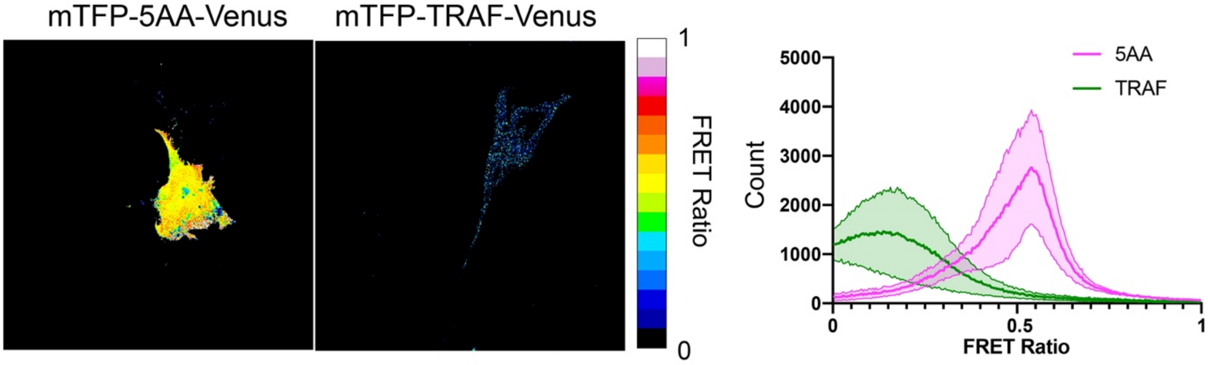

Supplementary Figure S7: FRET controls. mTFP-5AA-Venus and mTFP-TRAF-Venus were used to determine the dynamic range of the RhoA-biosensor.

Supplementary Figure S8:

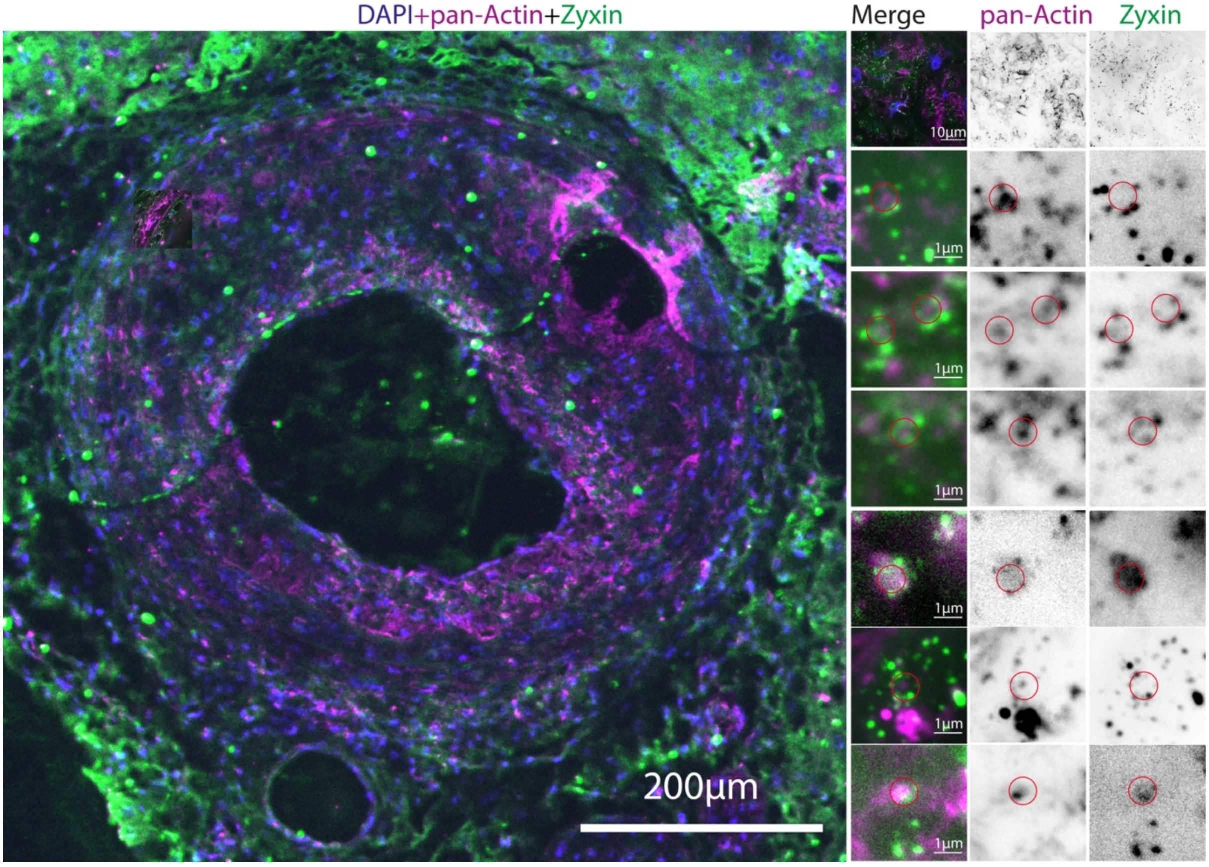

Supplementary Figure S8: Accumulation of actin and zyxin speckles in neointima of mouse arteries. Left panel shows an overview image of the artery after carotid ligation with clear evidence of neo-intima formation. Top panel on right shows a maximum intensity projection of a z-stack of the neo-intima, taken with a 100x Oil objective on a Nikon SoRa super-resolution spinning disc microscope with 2.8x SoRa magnification. The below panels show further zoom ins with examples of actin dots, surrounded by zyxin areas. Red circles indicate the typical 1µm podosome diameter.

Supplementary Figure S9:

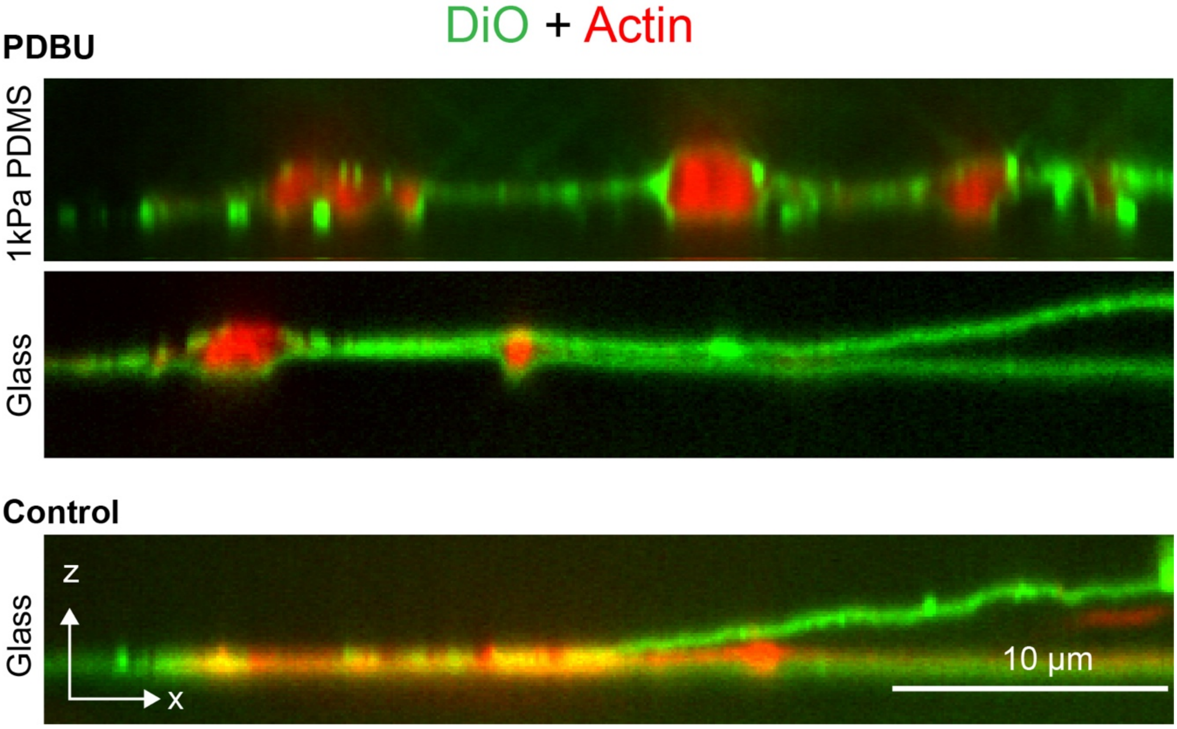

Supplementary Figure S9: XZ-projection of confocal image stack from A7r5 VSMCs on glass coverslips or 1kPa PDMS, stained with phalloidin and DiO. Podosome formation after PDBu treatment results in plasma membrane curving on PDMS and glass, which is absent in control cells with stress fibres.

Supplementary Movies:

Supplementary Movie 1:
PDBu stimulated vascular smooth muscle cell on 1kPa PDMS, expressing Tractin-Tomato. The images were taken at a rate of 30s per frame.

Supplementary Movie 2:
PDBu stimulated vascular smooth muscle cell on 130kPa PDMS, expressing Tractin-Tomato. The images were taken at a rate of 30s per frame.

Supplementary Movie 3:
PDBu stimulated vascular smooth muscle cell, expressing a RhoA biosensor and iRFP-Lifeact. Donor and FRET channels were taken simultaneously, using a two camera setup at a rate of 5s per frame. iRFP images were taken every 20 frames (i.e. every 100s).

Supplementary Tables

Supplementary Table 1:
List of 1027 identified proteins from quantitative proteomic analysis including description, and associated GO terms and pathways.

Supplementary Table 2:
Pairwise comparison of quantified proteins from A7r5 VSMCs seeded on 1kPa PDMS and subjected to atmospheric pressure or cyclic HT pressure over 24h.

Supplementary Table 3:
Pairwise comparison of quantified proteins from A7r5 VSMCs seeded on 130kPa PDMS and subjected to atmospheric pressure or cyclic HT pressure over 24h.

Supplementary Table 4:
Pairwise comparison of quantified proteins from A7r5 VSMCs seeded on 1 or 130kPa PDMS and cultured inside the pressure stimulator at atmospheric pressure over 24h.

Supplementary Table 5:
Pairwise comparison of quantified proteins from A7r5 VSMCs seeded on 1 or 130kPa PDMS and subjected to cyclic HT pressure stimulation over 24h.
